# Intersections between heritage, multilingualism, and education: language acquisition in India

**DOI:** 10.3389/fnhum.2025.1538482

**Published:** 2025-10-21

**Authors:** Vaijayanthi M. Sarma

**Affiliations:** Department of Humanities and Social Sciences, Indian Institute of Technology Bombay, Mumbai, India

**Keywords:** heritage, multilingualism, education, acquisition, policy

## Abstract

India is a profoundly diverse nation-state where constitutional provisions for two official and 22 Scheduled languages overlay a vast substratum of numerous Non-Scheduled languages and over a thousand distinct mother tongues, creating a rich and layered linguistic hierarchy. The trajectory of linguistic growth of young Indians is invariably multilingual and multidialectal and involves at least one L1, followed most often by Hindi and English or other languages. We carried out a qualitative study with over over a thousand students entering university using a modified LEAP questionnaire for self-assessment of fluency, literacy, domain of use, and time course of language acquisition and loss. We explore the interaction between heritage language, multilingualism, and the formal (trilingual) education policy and show that they intersect to redraw the linguistic profiles of individuals with shifting language dominance, and impact linguistic ability, especially in L1. We also find that the understanding of “heritage language” needs to be more nuanced in this particular context of multilingualism and language acquisition.

## 1 Introduction

The linguistic landscape of India is profoundly complex and diverse. This rich multilingualism, however, presents significant challenges for language maintenance and education, often leading to shifting patterns of language dominance and the risk of attrition for an individual’s first language (L1). While the trajectory of language acquisition for young Indians is invariably multilingual, the linguistic experiences and profiles are heavily shaped by the formal education system. The primary aim of this study is to investigate the changing patterns of linguistic competencies. We explore the complex interplay between heritage language, multilingualism, and formal education policy through a large-scale, self-reporting survey of over a thousand first-year university students. We discuss how these factors intersect to reshape the linguistic profiles, often with significant impact on L1 proficiency. A second and related goal of the paper is to nuance the understanding of Heritage Language (HL) within the unique context of Indian multilingualism, which differs significantly from migration-induced multilingualism that is typically studied as HL in many Western contexts.

The linguistic diversity is very straightforwardly demonstrated through the 2011 decennial census of India^1^ (CoI, accessible through the Census Digital Library) GOI (2011), which recorded 121 languages, including 22 Scheduled languages and 99 Non-Scheduled languages (see CoI statements 1, 2, 4, and 5). The census lists 270 identifiable “mother tongues”^2^ or L1 with 10,000 or more speakers each, of which 123 mother tongues are grouped under the Scheduled Languages (Part A) and 147 mother tongues are grouped under the Non-Scheduled languages (Part B). Scheduled languages are those that are listed in the 8th Schedule of the Indian Constitution (Articles 344(1) and 350 (GOI, 2024)). These languages are officially recognized, receive certain constitutional protections, and are supported by the government. The identified mother tongues or L1 with fewer than 10,000 speakers each but classified under a particular language were grouped under “Others”. The data show that 96.71% of Indians have one of the Scheduled Languages (see Appendix 1, CoI Statement Table 4) as their mother tongue and that approximately 3.29% of the population, which is a sizeable number in population terms, speaks languages that are not officially recognized. India’s population, according to the CoI, was 1.21 billion, with a third in urban areas and the rest in rural areas. Other facts noted by the census are that 26% of Indians are bilingual and 7% are trilingual, implying that almost two-thirds of the population may be monolingual^3^. According to UNESCO (2009), 6.18% of the world’s languages are spoken in India.^4^ The Greenberg diversity index is 0.930 (UNESCO, 2009, Table 7, p. 305^5^), which means that in 93% of cases, two randomly selected people in India will have different native languages. Hindi is numerically the most commonly spoken primary language in India, with an estimated 43.63% of the population speaking it as their L1.^6^ English is the second-most widely spoken language after Hindi, with an estimated 12% of the population being able to speak it, more commonly as L2 or L3, but only about 0.02% claim it as their L1. In the EF English Proficiency Index published by EF Education First^7^, India ranks 69 out of 116 countries with a score of 490, which indicates moderate proficiency but shows declining proficiency in recent years.

The language profile of India is complex not just nationally. The States Reorganization Act of 1956 (almost a decade after Independence) organized India along linguistic lines into 14 states. Several more states were formed subsequently through similar logic, raising the numbers to the current 28. This reorganization was not only an acknowledgment of India’s linguistic diversity but also sought to promote regional languages and cultures and ensure better regional governance. Each state has an official language and may choose one of the two official languages when dealing with the central government.^8^ The intra-state linguistic structures are also highly diverse, reflecting the presence of numerous minority languages as well as significant populations from other states that speak their own L1s, especially in larger metros. Internal migration is extensive and unrestricted. This diversity presents distinct challenges for educational policies within the states as well.

In this context of spoken language diversity^9^, primary, secondary, and tertiary education build on linguistic policies to varying degrees (Annamalai, 2001; Mohanty, 2012, 2018; Sarma, 2020; Sharma, 2001). Education is both a state and a central government mandate and, as such, most school education promotes the trilingual education policy (or the Three Language Formula) with a shift to English education in many tertiary institutions.^10^ The new National Education Policy (NEP, GOI, 2020) lays emphasis on early education in local or locally dominant languages, but that is unlikely to ease the pressure on minority languages; neither will it reduce the “global” aspirations via English. As Mohanty (2012) and (Fishman, 2001) note, language in education has a serious impact on language maintenance, and education policies impact the maintenance of linguistic diversity. Mohanty states that there has been a sharp decline over time in the availability of regional languages as the medium of instruction (MoI); where in 1970, 81 languages were used as MoIs, the numbers shrank by half to 41 three decades later, and then by another 25%, such that currently only 31 languages survive as MoIs in early education (up to grade 5, 10 years of age), 21 in the later school years (grades 7 to 9, 12–14 years of age), and 18 in the last 2 years of high school (15–17 years of age). English has a dominant presence at all levels and is almost exclusively used as MoI in most higher education institutions (HEIs), especially in the prestigious technical, professional, and science-based HEIs such as the one where this study was carried out.^11^

Many researchers (Annamalai, 2001; Bhattacharya, 2022; Bhattacharya and Mohanty, 2021; Gambhir, 2008; Koul and Devaki, 2001; Mohanty, 2012, 2018; Pattanayak, 1981; Raj and Prakash, 2020) have raised key concerns with respect to (a) social justice, opportunities, and recognition for linguistic minorities, (b) the ineffective and weakly implemented linguistic policies in education, (c) the actual (unsatisfactory) educational outcomes of the languages learned, (d) the impact of colonial practices on language recognition with the power hierarchy that was and has been created between the official languages, the state languages, and the “vernaculars”, (e) the lack of mother tongue-based education, and (f) the consequences for linguistic diversity, where most languages of the country barely make an impact within formal education systems to the detriment of their speech communities. We note that a vast number of spoken languages fall by the wayside during, and sometimes even before, education, and even those that are learned as L1 are powerfully impacted by the two official languages.

The kinds of changes over time that are reported in the survey by the participants, including loss of L1, restrictions on the domains of its use, reduced exposure over time, and stated intergenerational differences, are suggestive of patterns typically found in studies of HL. Polinsky and Kagan offer two definitions of HL: a broad definition applicable to those with cultural connections to a language but with no actual linguistic competence in it, and a narrow one applicable to those whose L1 is the HL but who did not fully acquire it because “of the individual’s switch to another dominant language” (Polinsky and Kagan, 2007, p. 369). The authors concede that heritage speakers can show a range of competencies regarding speaking, from complete fluency to barely any ability to speak, in comparison to other language skills (Fishman, 2001). These HL competencies are also tied to age of exposure, the order in which the languages are acquired (L1 before L2/L3 or L1 and L2 at the same time), the quantity of language exposure and whether it is sustained, and the generational status (in the context of migration to other linguistic areas) (Benmamoun et al., 2010; Cho, 2000; Cook, 2003; Polinsky, 1997, 2006, 2018; Schmid, 2011; Wiley, 2001). Montrul (2008) shows that core grammar is likely to develop more fully when there is early and sustained input and in a sequential fashion, but non-core properties may not develop as easily and require prolonged exposure beyond the early years.

In the Indian linguistic context, we find all these properties reflected in various ways: language dominance relationships determine exposure, cultural connections and family settings determine L1 acquisition, and education policies impact the languages learned prior to the commencement of formal education. However, these properties do not fully capture the shifting nature of the linguistic capacities of the speakers in the study. As we will see, even while the cultural connections are strong and exposure to non-dominant languages is continuous, the Indian context is quite different from the other cultural contexts where HLs have been explored. Crucially, many of the languages that show attrition, including the Scheduled and Non-Scheduled languages, are not in a wholly asymmetric power relation with the dominant languages, i.e., lacking a national identity, significant populations that speak them, cultural rootedness, literary traditions, or foreign geographies, and yet we find them showing similar effacement in their homeland. We return to these differences in the analysis section.

The education system of India is as complex a phenomenon^12^ as its linguistic composition, and we provide a brief outline here. The average student who arrives at a tertiary institution in India has typically received 14 years of school education. The Three Language Formula (TLF), as outlined in the National Policy Resolution (Ministry of Education, GOI), provides for the study of “Hindi, English, and a modern Indian language (preferably one of the southern languages) in the Hindi-speaking states, and Hindi, English, and the regional language in the non-Hindi-speaking states” (Ministry of Human Resource and Development, GOI, 1968). This grassroots approach to multilingualism has, over time, shown itself to be somewhat romantic in its imagination, and the divide that it sought to bridge between Hindi-accepting and Hindi-resistant states remains an unfulfilled goal. Since each state may have its own official language and differ in its dealings with the Centre (using either Hindi or English), the nature of linguistic exposure varies across states and school types. In India, there are municipal schools run by each state that tend to privilege the official state language as the medium of instruction (MoI), public schools of the colonial variety that foreground English as the MoI, centrally managed schools such as Kendriya Vidyalaya and Navodaya Vidyalaya^13^ that adhere to the TLF in letter (if not in spirit), and many private schools that may cater to L1, English, or Hindi as MoIs depending on demand and with an eye on profit margins. For the most part, the content of the curriculum is fixed through a national curriculum^14^ with some variations per state in the content and in the textbooks created.^15^ Schools across India may then have English (or Hindi) as a first, second, or third language, mixed with regional languages, Sanskrit, Arabic, or Urdu, or a foreign language such as German or French.

The most recent Eighth All India School Education Survey (8th AISES, 2009) finds the following patterns with respect to MoIs (see Table 1). The number of schools having primary (6–8 years of age), upper primary (9–12 years of age), secondary (13–15 years of age), and higher secondary (16–17 years of age) stages in education increased by 20, 40, 33, and 42 percent, respectively, since the 7th AISES in 2002. With respect to MoI, it finds that 86.62% of schools at the primary stage teach through L1 (a decrease of 5% since 2002). A comparison of rural and urban schools showed that MoI is the same as the mother tongue (MT) in 87.56% of schools in rural areas and 80.99% of schools in urban areas, compared to 90.39 and 92.39% of schools, respectively, in the 7th AISES. This shows an overall dominance of the MT in both spaces, but with a sharp decline in the use of regional languages, particularly in urban locations. In addition, the survey also shows that Hindi is quite dominant in use in the primary years compared to English as the MoI.^16^

**Table 1:** Medium of instruction in primary schools (based on 8th AISES).

Category	Percentage (%)
Rural primary schools using mother tongue	87.56
Urban primary schools using mother tongue	80.99
Implied percentage of rural primary schools using other mediums	12.44
Implied percentage of urban primary schools using other mediums	19.01
National percentage of primary schools using Hindi	51
National percentage of primary schools using English	15

The outcomes of high school language education are therefore far from uniform.^17^ Most students begin by acquiring a mother tongue at home, and when they begin formal schooling at the age of 3–4 years^18^, they encounter a second language often different from their L1. At this stage, many begin with one of the two dominant languages, English or Hindi, as the medium of instruction (MoI) in school and encounter a “second” language in formal terms, which may also be either English or Hindi or a regional language (which may or may not be their L1). Some are educated in their primary school years in their L1 and encounter another language a little later. In terms of language experience, there is quite a wide range of competencies across students. Some students will naturally begin with Hindi as their L1, learn Hindi as L2 with English as the MoI in school. Alternatively, they may learn English as L2 with Hindi as the MoI. This is the least diverse of the linguistic profiles, with two languages. The more common patterns are an L1 that is different from both Hindi and English, and an L1 and an L2 together with one of either Hindi or English. While there are clear rural–urban and socioeconomic differences, these impact the quality of outcomes at school more than the actual language exposure patterns. It is seldom the case that there is a change in what is declared as the L1 or a change to the linguistic identity of the student, and neither is there attrition or diminution of the cultural or familial connections, such as we see in studies on HL generally. A second purpose of this survey, then, is to attempt to nuance our understanding of HL within the Indian context of multilingualism, which is quite different from, primarily, migration-induced multilingualism in other countries. In the following, we begin with the Methods in Section 2, followed by our Analysis in Section 3, and the Discussion in Section 4.

## 2 Methods

The survey’s main aim was to obtain a language profile for each student and to correlate it with their actual and perceived needs during their tenure at the institution. A secondary purpose was to follow the self-reported changes to their linguistic profiles over the course of their education. Given that the core outcome was to identify those who were in greatest need of improvement in the MoI, which is English, and in order to better understand the linguistic competencies of the students entering the HEI, we conducted a survey. There were 1,375 responses in all, with a declared sex ratio of 1:4, comprising 277 female students and 1,098 male students.^19^ The survey questionnaire was an online form to be completed while seated in an exam hall before an app-based English proficiency test^20^ was administered to determine proficiency levels. We used the Common European Framework of Reference for Languages (Council of Europe, 2001), which is an international standard that defines language proficiency across six levels: A1 (Beginner) users can grasp basic phrases (A0 is an absolute beginner), while A2 (Elementary) users understand simple sentences and common expressions; B1 (Intermediate) level individuals can comprehend main points in familiar contexts; B2 (Upper Intermediate) users can understand complex texts and technical discussions; C1 (Advanced) users can understand a wide range of demanding texts and implicit meanings; and C2 (Proficiency) users can effortlessly understand and summarize information from various sources. A minimum of a high B1 or a B2 level is useful for academic purposes at an HEI.

For internal pedagogical purposes, all the participants were also asked to write a short passage in English on a topic of their choosing from among several that were given. The written work was used for the following two purposes. First, there are significant variations within the CEFR levels for the language skills that the test evaluates and a composite score is not fine enough to capture the differences. The essays allowed us to augment the evaluation of comprehension, expression, and productive writing, often the hardest skills to acquire and which the app based test could not do. Second, scores are often clustered at the borders between levels and the essays served as a secondary check. The attention was not so much on the lack of lexical range, use of idioms, punctuation, spelling, flair, and such, but more on the ability to express complex ideas and the ability to build on those ideas in a clear and comprehensible way. While these assessments enabled the development of materials and lessons around key linguistic concepts that needed to be addressed, the written work is not in the scope of this study. We limit ourselves to noting that the students’ expressive writing reflected their self-assessment in English. This measurement is conducted to provide additional English language support to those who require it, since the MoI at the institution is English.^21^

The questionnaire (see Appendix 2) was an adapted version of the Language Experience and Proficiency Questionnaire (LEAP-Q, Kaushanskaya et al., 2019; Marian et al., 2007) and excluded questions that were irrelevant to the context or sought information that was already known (such as cultural connections, years of schooling, immigration, disability, accent) and was focused on the linguistic skills. Demographic data was also not sought, as we explain later. We used the adapted LEAP-Q as it is a widely accepted instrument designed for gathering self-reported data on language experience in bi-, tri-, or multilingual populations and a standardized tool that could be used for this type of survey. While the survey relies on self-reporting as the main means of assessing proficiency in languages other than English, research has shown that self-assessment can be a valuable tool. Some studies have shown a high correlation between self-reported proficiency and objective test scores (Bachman and Palmer, 1989; Freeman, 2000), even though such reporting may be affected by or vary from direct assessment of language abilities for various reasons, such as the context of acquisition, the specific language skills being assessed (e.g., speaking vs. reading), and the dominant languages of the participants or other factors (MacIntyre et al., 1997). While we did not correlate this self-reporting with other direct testing measures (a near impossibility given the number of languages—about 27—that are known), and this remains a report at one point in time of the individual linguistic trajectories, we find that this questionnaire allows us to efficiently gather data on language acquisition, use, and reported proficiency across a large and linguistically diverse population and offers us an interesting and comprehensive account of how linguistic competencies have been shaped by the language policies and education system, which was the primary aim of the study. Objective assessment of individual language skills remains a challenge in this context of wide variation and a possible avenue for future work.^23^

The questionnaire sought self-assessment and self-reporting on questions such as the following: (a) the list of languages known in order of dominance of use, (b) the exposure they currently receive to each of those languages, (c) preferred language for reading texts, (d) preferred language for speaking and conversation, (e) order of acquisition of the languages known, (f) the language most frequently used in schools with teachers, (g) the language in which subjects like history and geography were taught and the language in which science and mathematics were taught, (h) the third language learned formally in school, if any, and (i) whether, in their estimation, they had forgotten any language(s).

These were followed by a specific subset of questions for each of English, Hindi, and the regional language that they speak the most (which, for about a third of the cohort, was Hindi and the associated languages as grouped in the census). Within these sections, we sought feedback on (a) age of acquisition, (b) age of gaining fluency in speaking, (c) age of beginning literacy, (d) age of gaining fluency in reading, (e) simple scale measures of fluency in speaking, (f) simple scale measures of comfort with reading instructions or filling out forms, and (g) the domains of use of each of these languages. The questionnaire ended by asking the students if they sought any language-specific help from the institution.

The questionnaire was administered in English, which is the language used in all formal and academic contexts at the HEI, and students at this level of education were accustomed to responding to forms in English. Teaching Assistants were available to assist. The questions were tractable and the students had no particular difficulty in answering the questions.

## 3 Analysis

We begin by presenting the large-scale patterns in the responses and then move to the finer details of the linguistic experiences that we have gathered from the survey. Unlike the national census, where only a third of the population was bilingual or trilingual and the rest primarily monolingual, we found that about 23% of the participants were bilingual (Hindi and English), while the rest, a dominant 77%, were trilingual (with Hindi, English, and a regional language as the L1). This was expected because most students have gone through an education system for ten to twelve years with the TLF.

A total of 27 unique languages were listed in the survey as “known” languages, including all 22 of the languages in the 8th Schedule of the Constitution. The dominant mother tongue was Hindi and a few variants (23%), but this again did not approach the national counts, as may be expected, since the participants are drawn from all areas of the country. The mother tongues were primarily one of the Scheduled languages, such as Marathi, Gujarati, Bengali, Rajasthani, Marwari, Tamil, Telugu, Kannada, Malayalam, and Odia, along with a smattering of others like Assamese, Punjabi, Sindhi, and Tulu. Only very few spoke (or had acquired) a Non-Scheduled language as a mother tongue. This is both a matter of some concern and a matter of statistics; many students from minority language backgrounds do not have access or are unable to access HEIs through the very competitive national entrance exam processes. Often, such linguistic backgrounds go hand-in-hand with other kinds of social marginalization. Equally, as a ratio of the country’s population the numbers of such students are small in comparison to the numbers of students speaking the more populous and dominant regional languages.

Marathi and Hindi accounted for the mother tongue of almost 45% of the participants, Telugu (the only dominant Dravidian language) for about 12%, and Gujarati, Rajasthani, Marwari, and Sindhi (the other Western Indian languages) for about 13%. The reasons for this profile are not entirely linguistic. The competitive exam has a feeder system of “coaching” or exam preparation centres. This is external to the school system and is itself a barrier to more equal representation. A second factor is the location of the students’ homes, which also influences the selection of the HEI. In terms of usage dominance, Hindi and English were deemed to be the most dominant. However, when a regional language was provided as one of the languages used, the dominance of Hindi or English was mediated by geography—with English being more dominant when a Dravidian language was provided as the regional language, and Hindi being more dominant when another Indo-Aryan language was provided as the regional language.^24^

**Figure 1:**
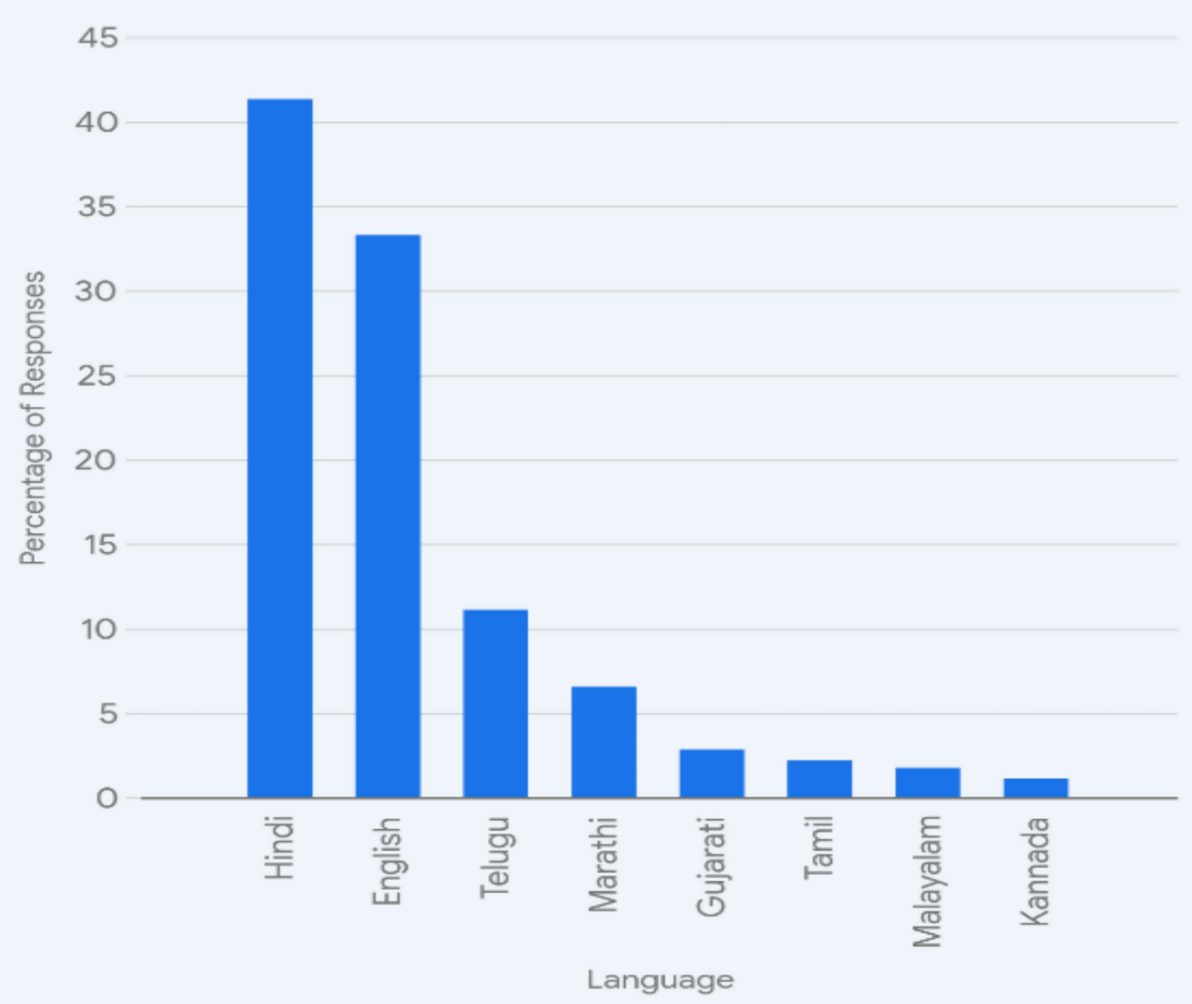
Percentage of dominant languages.

As visualized in Figure 1, Hindi and English are the dominant languages for almost 75% of the students. Given the practical need to also communicate with students from other regions, we expect the two national languages to crowd out the others. While there is some use of regional languages like Marathi (6.5%), Gujarati (2.8%), and Telugu (11.1%) given the overall percentage of Telugu speakers, very few of the unique languages mentioned in the responses accounted for the overall dominance.^25^

**Figure 2:**
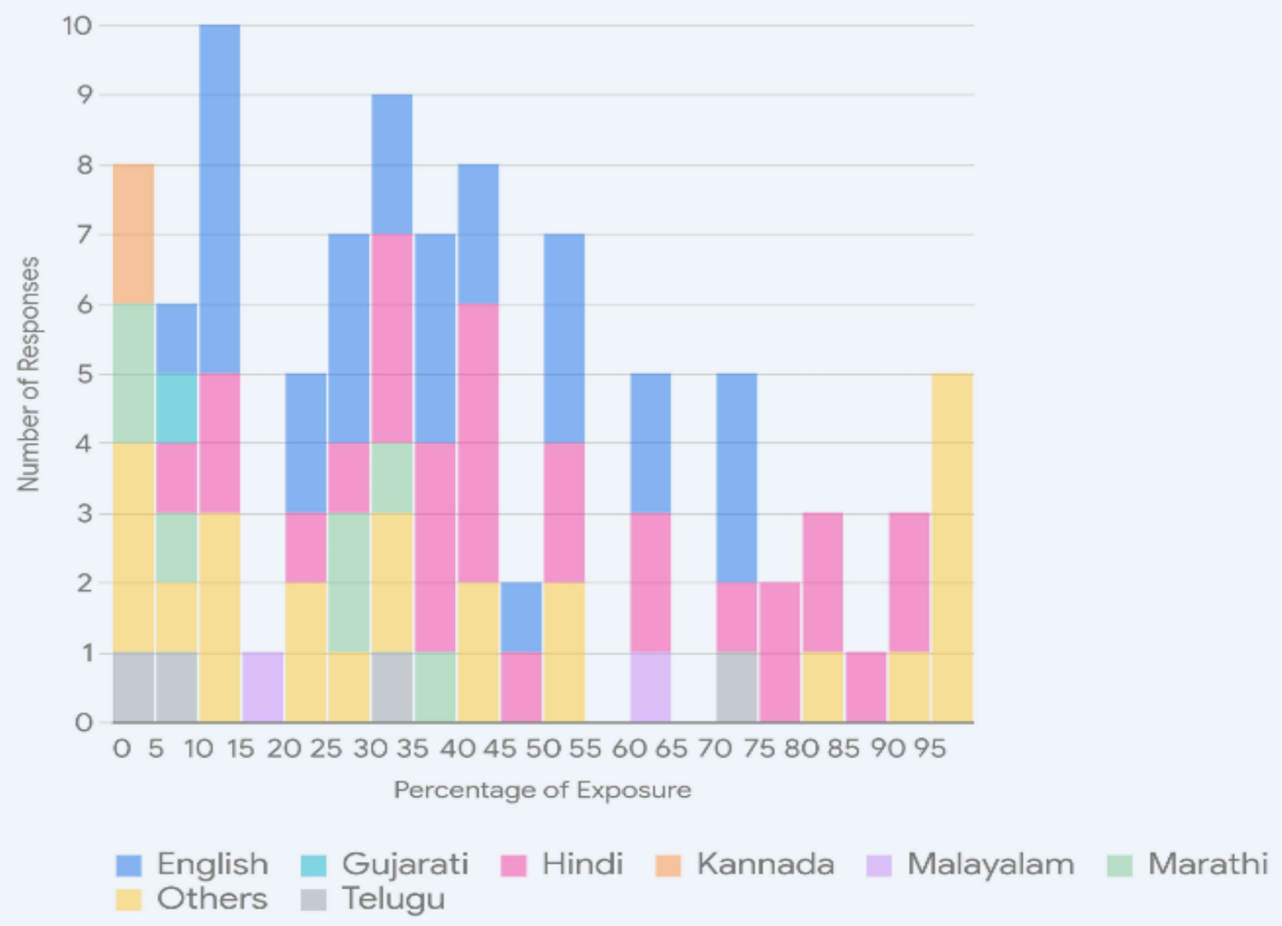
Overlaid histogram of language exposure.

The responses to the question asking about current (continued^26^) exposure to the languages showed similar patterns to the overall dominance. In Figure 2, the overlaid histogram provides a summary overview showing the distribution of language exposure percentages for English, Hindi, Marathi, Tamil, Telugu, Malayalam, Gujarati, Kannada, Sindhi, Rajasthani, and Marwari. The remaining languages have been assigned much lower percentages and are grouped under “Others.” The percentages represent the quantum of exposure across languages (colour-coded). For example, English receives exposure across the responses between 5 and 75%, Hindi between 5 and 90%, and so on. The histogram shows how widespread the two national languages are across individuals in contrast to the limited space claimed by the regional languages.

Hindi and English, again, received the greatest number of responses across a variety of percentages and were used widely. The following patterns are most prominent and account for the greatest number of responses: (a) Hindi and English are used equally; Hindi is used relatively more often with English as a close second or the converse; so essentially only two languages are used; (b) Hindi and a regional language are used, followed by English; (c) English and a regional language are used, followed by Hindi. Only a very few (about four) said that they did not know one of Hindi or English. In these cases, the student had either been raised with Hindi alone through school or had never seen Hindi and had grown up with an L1 and English as L2. Even when the students rated themselves as having had no English at all, there is enough competence for comprehension and for functional responses in the questionnaire, with some short text answers written in Hindi/Hinglish in the Roman script.^27^ A moderate number of responses were observed for Marathi, and the individual bars are relatively short in the image for the remaining languages (Tamil, Telugu, Malayalam, Gujarati, Kannada, Sindhi, Rajasthani, Marwari), indicating that these languages received both fewer responses and self-assessment at lower percentages of current exposure. The shifting dominance away from any L1 to Hindi and English is the pattern observed across cohorts as well^27^, underscoring the impact of education policies on linguistic proficiencies.

The next few questions were directed at understanding the impact of education on language, with emphasis on literacy. Given that the participants were bi-or trilingual, the questions elicited self-assessment of reading and writing in the languages they knew. Students were asked if a text (article, story or other) were to be made available in all the languages that they said they knew, in what language would they choose to read that text. Two thirds of the responses indicated a clear preference for English over any other language when it came to reading. This was a remarkable finding, though not a surprising one, that literacy centered on English rather than any of the other languages. Hindi was the second most preferred language for reading though significantly less so than English. The participants appeared to be best equipped for reading and writing and most literate in these two languages. This is contrary to Koul’s (2001) findings that we discussed earlier. Even classic or literary languages like Marathi, Gujarati, Tamil, Telugu, and Kannada were only mentioned with much lower percentages even by those speakers for whom the regional language was dominant.^28^ Thus, L1 or the regional language was not at all in competition, even when the language was associated with a strong literary culture. It also indicated that minority languages, absent strong literary moorings, were at even greater risk of attrition/loss of competence for their speakers.

Relatedly, over two-thirds of the participants (65%) used English as the dominant language when talking to their school teachers, and only Hindi and Marathi found any mention at all in this domain. Over 90% of the responders studied all their school subjects (social studies, geography, history and civics, and sciences including mathematics) in English. The remaining students showed varied patterns. Some were taught in English, but explanations were in the local language (seen especially with Hindi and Telugu), while some others learned Social Studies in Hindi, Marathi, or Telugu. The emphasis remains largely on English in school and can be directly correlated to the literacy skills in English and, to a lesser extent, Hindi.

We must clarify that, given the range of language options, schools may use the state language for teaching a few subjects, typically social studies, and during music, art, or physical education sessions. The state language may also be used throughout the day by the children among themselves, both inside and outside of class. L1 as MoI can also mean that the sciences are taught in L1, but this practice is on the wane because it is not integrated well enough with the later transition to HEIs that more dominantly teach in English. Thus, the exposure to the languages learned may be limited to just the specific class time or may permeate both in-class and outside-class hours. When languages are taught only in the language class, they are very poorly learned, and this is the outcome that we found most often with the third language. Access to the language in the family context and in the larger community also helps with fluency and maintenance of a language. In this context, socio-economic and geographical locations become relevant. Borooah and Sabharwal (2022) point out that studying in Hindi or a regional language appears to restrict the range of subjects that can be studied in the HEIs to mainly Humanities, while studying in English expands the subject availability to the sciences, commerce, engineering, medicine, management, and such, which also have better employment opportunities (their Tables 1, 2, p. 6). The observed restriction probably follows from having more HEIs in the “technical” subjects teaching in English, which may keep out students who have been primarily educated without English, and also from HEIs teaching in regional languages/Hindi not having the capacity to deliver education in these other subject areas and finding themselves limited to the classical subjects in the Humanities. This restriction–expansion, as we said, also plays into the socio-economic-geographical divides.

A final question in the context of education and linguistic experience concerns the application of the TLF. Typically, most education boards have prescribed a third language to be learned between grades 5 and 8 (middle school, 10–13 years of age). Some systems allow for a different language in grades 1–4 (primary school, 6–9 years of age) and transition to yet another in the middle school years. This selection is determined partly by the state policy on learning the official language of the state. A few boards of education and a few states have removed this requirement altogether.^29^

**Figure 3:**
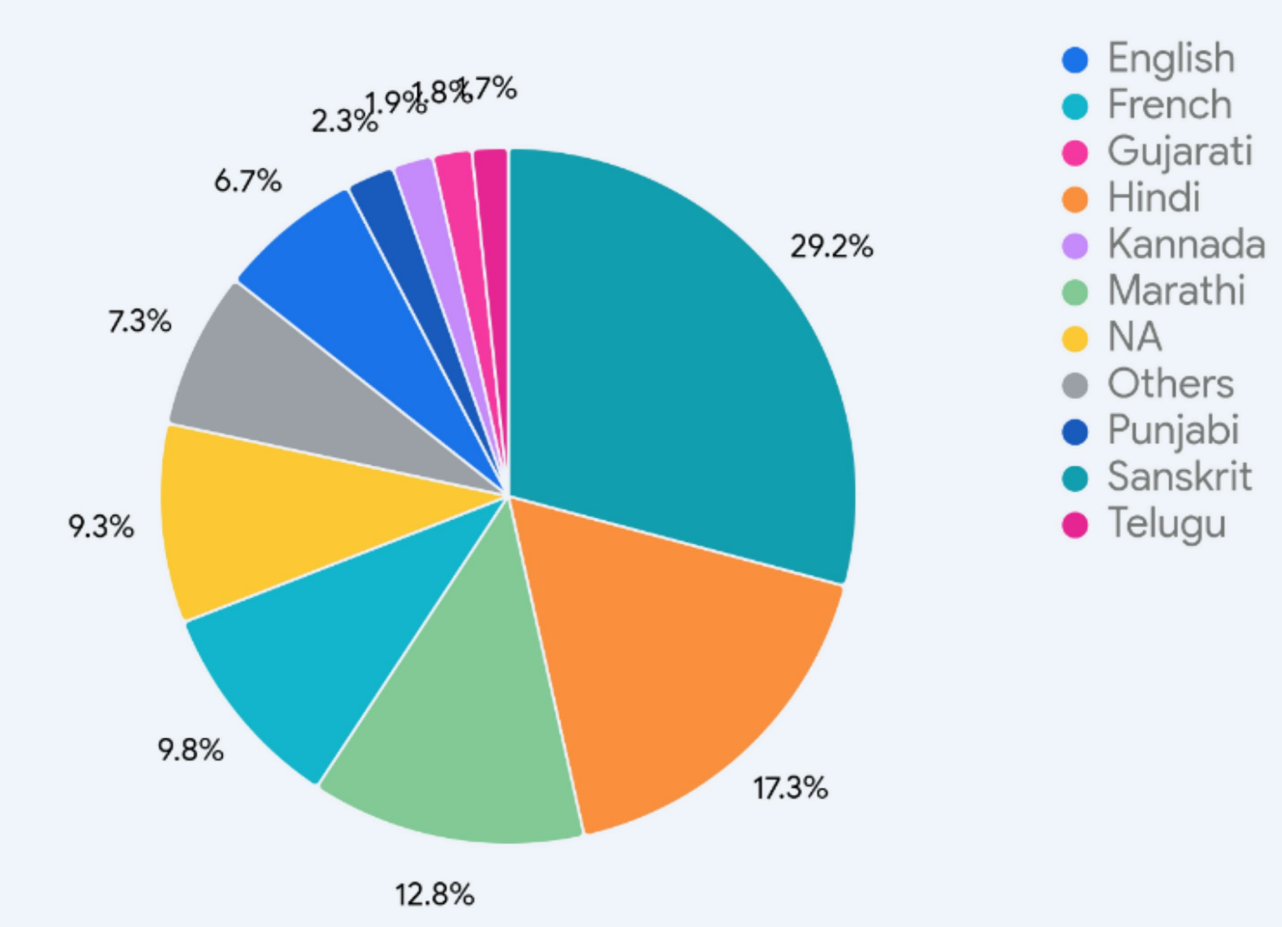
Distribution of third languages.

Figure 3 shows the percentage distribution of the languages that were learned as a third language. What was noteworthy was that Sanskrit was the most common third language that was learned, followed by Hindi, Marathi, and French. A primary motivation in the selection of Sanskrit is its similarity to Hindi, with no new script to be learned and a large degree of lexical similarity, which makes learning appear less burdensome on the face of it.^30^ A secondary motivator appears to be the ability to score well in the exams. About 7% learned English as a third language, and about 9% did not learn a third language at all.^31^ In the context of L1, very few students learned one of the mother tongues or even a Scheduled language as a third language. With fewer than 20 responses, they were grouped under the label “Others” and included only 7% of the students but over 20 unique languages, including Arabic, Assamese, Bengali, Bhojpuri, Kannada, Chhattisgarhi, Dogri, Garhwali, Hadoti, Kashmiri, Konkani, Kumaoni, Maithili, Malayalam, Manipuri, Marathi, Nepali, Odia, Punjabi, Sindhi, Tamil, Telugu, and Urdu. Many of these languages have a large and stable population of speakers (that is, they are reasonably placed on the EGIDS^32^ scale at the macro level, EGIDS 1–4), but the representation within the education system that is building towards higher education allows for little emphasis on them. As we will see, this observation also factors into the contexts of use of the various languages; for an educated, mobile cohort, L1 is, in effect, being pushed down the scale at an *individual* or *micro-community* level (to a Level 6—In Trouble, or even Level 7—Shifting: Child-bearing generation can use the language but are not transmitting it).

In direct contrast to issues of literacy and the education system, we observed the opposite pattern of self-assessment for conversation and speaking. When the interlocutor could speak all the languages known to the responder, we found that the regional languages, including Hindi, have a distinct advantage. When Hindi is the L1, it tends to dominate spoken language contexts. Thirty-one percent of the students also said that they used English. Nonetheless, the use of the L1 is much more established in spoken/aural contexts with peers than in reading and writing. The somewhat higher evaluation for English quite likely follows from a significant amount of code mixing and code switching between the L1 and English.^33^

When asked about the order in which they had acquired/learned the languages they knew, the primary answer was almost always Hindi or a regional language. Less than 5% listed two languages before the age of 4, indicating that most of them were raised with one dominant language and began to learn the other(s) as they entered the formal school years. We may recall that the participants’ language profile indicated they were bilingual or trilingual. This suggests that most were sequential bilinguals.^34^ On average, the age of acquisition of Hindi and the regional languages began at birth, with self-assessment of spoken language fluency averaging at 5.5 years of age. Reading skills for Hindi correlate with the school-going age of about 5–6 years, with fluency being gained on average during the primary school years at around 9 years of age. In contrast, reading skills for the regional language were rarely attained, and when fluency was reported, the ability showed rapid decay as well. The average age at which English was acquired was well into the school years, averaging at about 6.8 years, with full fluency in speaking (11.5 years) and reading (10.3 years) being achieved around the middle school years. It may be noted that the self-assessment of spoken language fluency was almost a year later than literacy skills. This can be attributed to an emphasis in schools on literacy (reading and writing) over spoken language skills.

We also asked the students to rate themselves on a scale of 1–10 for fluency in speaking and fluency in reading instructions or forms in Hindi, English, and the regional languages, and we found interesting patterns. We grouped all the regional languages together, given that only a very few of them were numerically dominant and all function along similar lines for fluency ratings.

**Figure 4:**
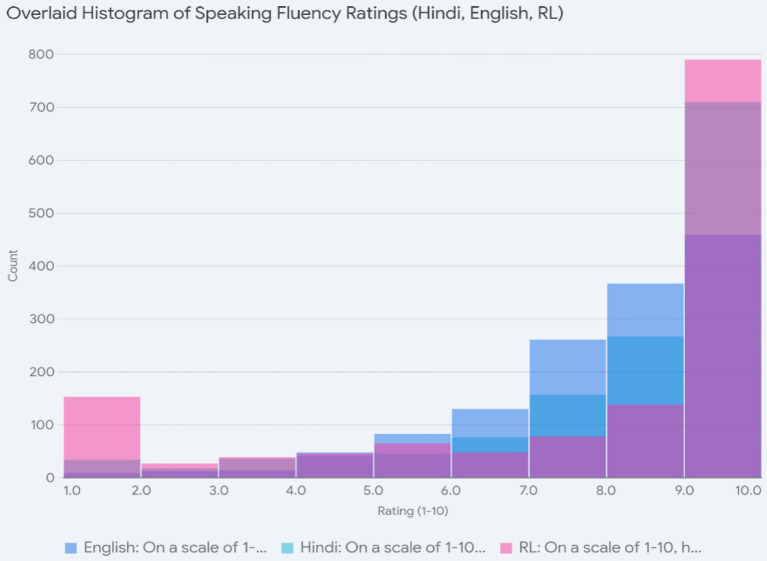
Speaking fluency ratings.

In Figure 4, the overlaid histogram presenting the comparative scores for Hindi shows that self-assessment of fluency in spoken Hindi is towards the higher end of the scale, with a large number of participants rating themselves at 9 or 10, indicating a high degree of comfort in the language. Very few rated themselves lower on the scale, and such self-ratings were typically from the very few participants who learned Hindi as a third language. The ratings in English also tended towards the higher end of the scale (7–8) but were not as high as those for Hindi and occurred in a more spread-out fashion. Several students rated themselves as moderately fluent (i.e., at the middle of the scale) in English, indicating that the degree of comfort with English was less robust than with Hindi. This can be correlated with the age of learning English and its being used less as a mother tongue and more as a means for formal education. Finally, the self-assessment for speaking the regional language was different from both the others and interesting in that it had peaks at both ends of the scale and very little in the middle. As with Hindi, a large number declared themselves to be very fluent in the language (native speakers), and an equally substantial number reported low fluency. We will return to this pattern in our discussion since it tells us about HLs in India. We gathered that both Hindi and English were widely used and learned, while the other L1s either stayed with the learners or were completely lost—there seems to be no middle ground that they occupy. In other words, L1 is becoming less ‘salient’ and requires more cognitive effort to access in certain domains, pushing them away from intermediate fluency.

**Figure 5:**
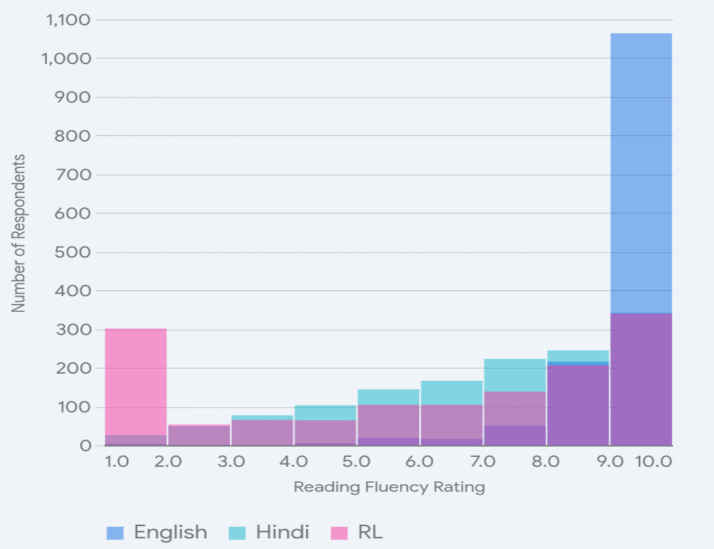
Distribution of reading fluency ratings across languages.

The preceding outcomes can be compared to self-assessment for reading scores, as shown in Figure 5. The ratings for English and Hindi presented quite the opposite picture for reading compared to speaking. The scores for English were now heavily weighted towards the higher end of the scale, with many students rating themselves at 9–10. Reading proficiency in English was quite high compared to the more distributed evaluation of spoken English abilities. The distribution of Hindi, by contrast, was spread out, ranging from the middle of the scale upwards, with many assessing themselves as poor readers in Hindi (self-assessment scores of 2–3). The distribution for the regional languages was more diverse than the other two and different from the spoken language assessment. While there were participants who rated themselves at the two extremes of the scale, there was a large spread of responses. Some of this is likely related to language instruction in school in the first language, the cultural connections they feel, and the literary heritage of the languages they identify with.

**Figure 6:**
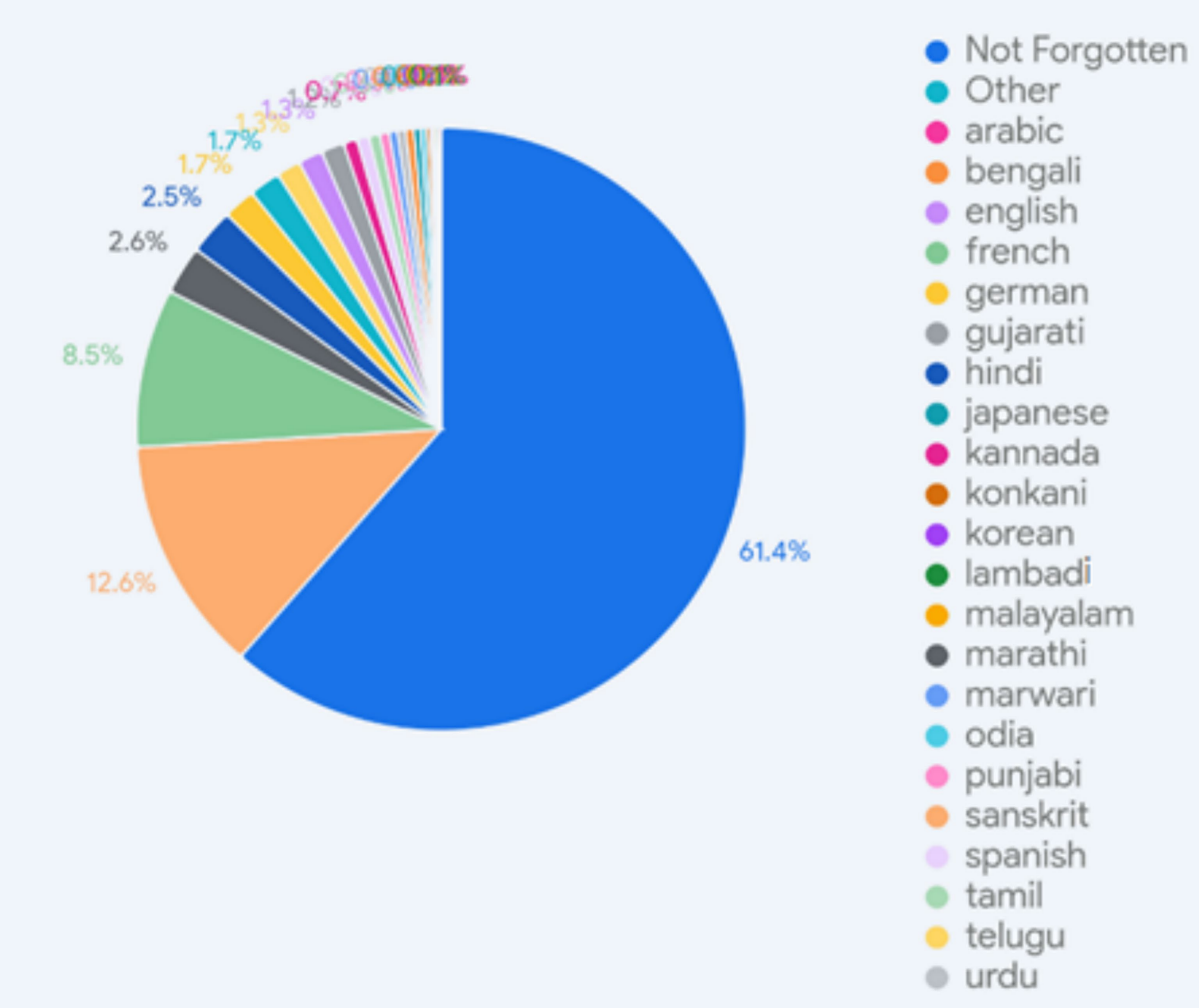
Distribution of forgotten languages.

In the context of multilingualism and changing patterns of use, we asked the students if they had forgotten any of the languages they had acquired or learned. The data are presented in Figure 6. A majority of the students (61%) said that they had not forgotten any of the languages they knew (which included a large number of Hindi–English bilinguals), while the rest (about 39%) indicated some language loss. The kind of loss also varied from a loss of writing skills (forgetting the script) and some lexical attrition to a complete loss. Unsurprisingly, the most frequently forgotten languages were those acquired in school, formally, as a third language. Sanskrit, the third language that is most frequently learned (29%), was the most frequently mentioned forgotten language (12.6%). Other languages mentioned include French, Spanish, German, Hindi, Marathi, Telugu, Gujarati, Kannada, Tamil, Marwari, Urdu, Punjabi, including many potential L1s. The regional language that was acquired was often the one lost, apart from the languages that were learned merely in a classroom, and comprised almost 10% of the responses. Language attrition over time was significant but not limited to L1 that was acquired first. There seemed to be a mixed pattern arising as much from a lack of functional relevance as from a lack of sustained exposure, which is critical to language maintenance. The TLF, then, is not in itself adequate to ensure retention.

Multilingualism invariably entails the use of languages in specified domains—the languages are not used equally frequently in all contexts of use (Annamalai, 2001; Gambhir, 2008; Kagan, 2005). We have already seen differences in patterns of fluency between spoken and reading skills. Given the substantial differences in the age of acquisition, dominance of language, and the language of formal education, we asked the participants to indicate the contexts of use of the languages that they knew, in order to better understand their linguistic trajectories.

**Figure 7:**
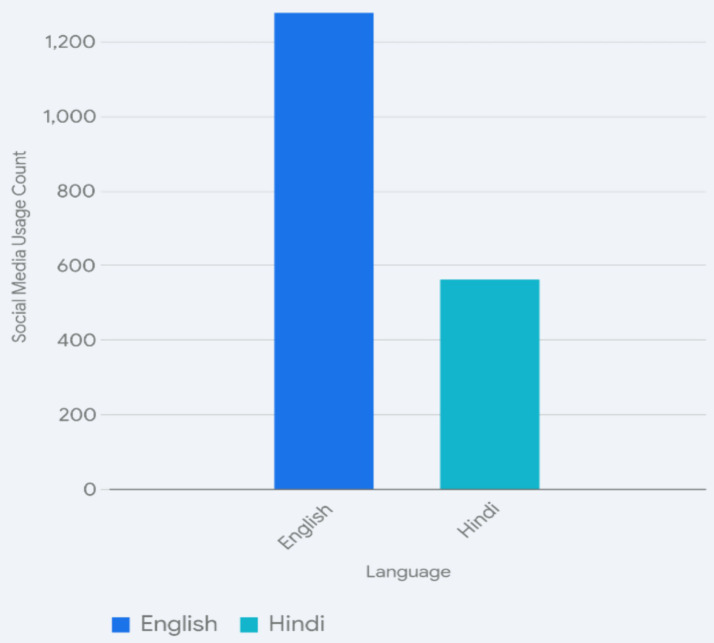
Social media use by language.

Hindi was primarily used for interpersonal communication, entertainment, and daily life. Students chose interacting with friends and younger family members (1,218 responses), watching TV programmes and movies, or listening to music and the news (1,109 responses each), and shopping or dealing with vendors and the larger society (1,061 responses) as the main contexts for the use of Hindi. In contrast, English was predominantly used for formal and casual reading (1,285 responses) and for activities online (such as engagement with social media, 1,276 responses), but also for casual reading, watching TV/movies, listening to music, and interacting with friends. The regional language was used to interact with family members, with an emphasis on older family members and grandparents (1,135 responses), interacting with friends, shopping, interacting with vendors and the larger society, watching TV/movies, listening to music, and using social media, but dramatically less so than in English. The responses suggest that the regional language is used for interactions with family and friends, social connections, and daily life within a local community. It is important to note that the students said they interacted with their older relatives in Hindi (53.12%) or in a regional language (46.88%) but never in English. This shows an intergenerational divide in language use, which may destine the regional languages to become HLs. However, it also tells us that English has not fully entered this domain of communication, though in urban areas we do find that this shift is increasingly visible, especially where the parents are “westernized” and already comfortable in English. In Figure 7, we can see that English outstrips Hindi as the popular language for social media and online presence.

The questionnaire ended by asking the participants if they sought any language-specific help from the institution during their tenure. The survey’s main goal was to obtain language profiles in order to assist those who needed help with the MoI, given the varied socio-economic, geographical, and linguistic backgrounds. As expected, one primary concern was a lack of confidence in their ability to use English and particular challenges in spoken language skills. A subset of the participants had more specific but allied requests concerning language skills, such as improving pronunciation, grammar, or writing. Interestingly, some expressed a desire to learn other languages, suggesting an enthusiasm for increased multilingualism, not a lessening of it. While during the school years, language classes can be seen as a curricular burden, the students clearly did not have negative feelings about their multilinguality as a social circumstance. In the survey, many expressed regret at not having learned the languages well enough; i.e., the participants recognized the loss. However, the interest was not so much for various L1s or even the dominant regional language, but often for foreign languages such as German, Japanese, and Chinese. Some students articulated concerns that their introverted nature was an obstacle that made the use of expressive language difficult and sought assistance in negotiating communication situations. The desire to develop skills in English is most directly linked to concerns about professional placement (being able to handle interviews or discussions and finding jobs), but is also about general upward mobility and the need to assimilate into a workforce. Given the preceding analysis of the dominant properties of the linguistic profiles, we discuss the findings in the next section.

## 4 Discussion

The two main purposes of this study were to examine the coexistence of English-based education at the school and tertiary levels with other languages at the school level, to track how individual linguistic profiles change over time, and to understand literacy in contrast to overall language competencies. A second purpose of this survey was to understand HL in the context of Indian multilingualism and the unique features found here that differ from those typically discussed.

The the profiles of the participants in the current survey is not an unusual one. In most national HEIs, where the students are drawn from across the nation, the linguistic profiles are likely to be multilingual but tend to use Hindi or English dominantly, given the diversity of the student body, and express greater facility in English for reading and writing.^36^ Institutions that draw students from more local areas will show a greater dominance of the regional language together with English and/or Hindi. These patterns provide insights into the linguistic landscape in India.

The questionnaire collected language experience data, but other demographic data such as stated religion, community, socio-economic (class and caste), or geographical locations (urban–rural divide) were not sought. This was partly to ensure privacy, but also because these factors, especially the socio-economic ones, may affect the “quality” of linguistic exposure but not the “types” of exposure directly (as was also outlined earlier in our discussion of education). Borooah and Sabharwal (2022) shed light on these factors through their analysis of Indian National Sample Survey Office (NSSO) data, which provide specific information on education from the 71st Round (January–July 2014) and the 64th round (July 2007–June 2008). They examine the MoIs present at the Primary (age 6–10 years), Upper Primary (age 11–13 years), Secondary (age 14–15 years), and Higher Secondary (ages 16–17 years) stages at school, and during Higher Education (age 18–22 years), and present data for various socioeconomic groups, as well as for gender, poverty, and rural–urban divides. They note that the distribution of MoIs is approximately 20% English, 45% Hindi, and 35% regional language in the primary years with no significant gender differences. This distribution remains steady until Higher Secondary when “English as MoI jumped to 34% (from 21% at Secondary level) while the proportion of students studying in a regional language fell to 26 percent (from 37 percent at Secondary level), with the proportion of students studying in Hindi remaining largely unchanged at around 40 percent” (Borooah and Sabharwal, 2022, p. 11). Higher education saw a further increase in the proportion of students, with nearly half of all students (49%) studying in English. All these findings are congruent with ours.

**Figure 8:**
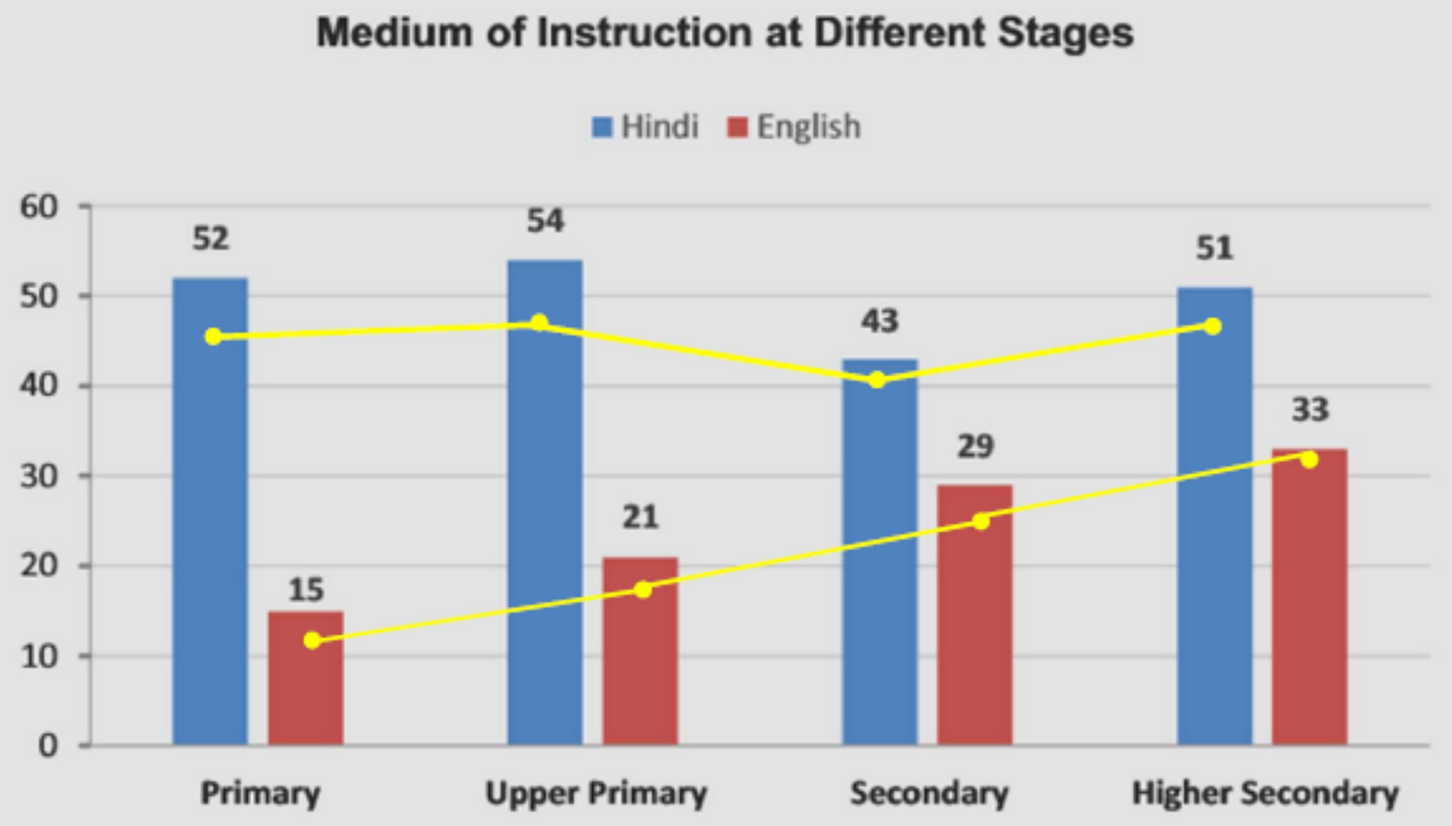
Changes in Hindi and English as MoIs during school (adapted from AISES 8).

Data in the AISES reports also capture similar patterns (Figure 8). The yellow line indicates the patterns from the 7th AISES (NCERT, 2009), showing overall growth for both languages. The report displays the change in MoI at various stages. Hindi is generally stable through the school years (the dip in the secondary years is not explained), but English shows steady growth over the school years. The same patterns of change are visible in both reports, except that the gross number of schools has increased over the years.

Borooah and Sabharwal (2022) show a geographical rural vs. urban divide as well as an economic poor vs. non-poor divide. English is much less available as MoI in rural areas compared to urban ones in their study. The percentage of students accessing HEIs in urban areas with English as MoI (66%) is almost twice that of rural areas (35%). However, the overall trajectory of increasing English availability is visible in both populations—across the school years, 13% of rural students had access to English as MoI in the primary school years while almost 49% did so in urban areas, growing to about 24% by higher secondary in rural areas and to about 53% in urban ones. The poverty divide also limits access to English for the poor, starting at 6% in the primary years, about 16% in the last years of school, and 28% in the HEIs. Aneesh et al. (2024) also use the NSSO data from 5 years, including the 74th round in 2017, and show that while rural areas have gained in many ways, there is still inequality of access. The AISES data differ from these in showing a comparable and fairly robust presence of MT (L1) education in urban and rural areas, especially in the primary school years (see Table 1).

Geographical and socio-economic locations can affect access, and these relations are fairly complex and hard to tease apart. We take the preceding studies as evidence that schools offering English as MoI are more numerous in urban areas than in rural ones and, correspondingly, that private schools, which are more abundantly present in urban areas, skew these numbers. We do not take this to reflect the “quality” of either language competence or education. The participants in this study are drawn from several distinct types of schools and from different parts of the country. What we do find is that the individual language trajectories remain comparable and move in directions that show us how education policies affect the nature of multilingualism.

The survey found that most participants were self-declared bi-or trilinguals, which is not typical of the national distribution, if the census is to be believed, but skewed by virtue of education. Most appear to be sequential bilinguals with adequate exposure to L1 until the age of 5 or 6, before exposure to Hindi and English began, when Hindi was not the L1. As may be expected, L1 fluency is attained earlier than with the two official languages. English entered the repertoire later, and fluency was also achieved later, with reading fluency preceding spoken language fluency by almost a year.^37^ It should be noted that when Hindi is the L1, there is still a considerable difference between the formal Hindi that is taught in school and the one learned at home. In fact, often students meant exactly that when they said they “forgot” Hindi, i.e., the attrition was of a version of formal Hindi and not the one used informally.

Montrul (2008) and Silva (2003), and others show that sequential bilinguals do better with core grammatical properties and the lexicon than do simultaneous bilinguals. This superiority is attributed to the quantum of exposure that the child receives in each language in a given period of time, which is (about) halved in the case of simultaneous bilinguals. A sequential bilingual receives more concentrated exposure at home and can build the grammar more fully and successfully. Given that the participants in the study were predominantly sequential bi-and trilinguals, it was also not surprising that over 60% say they had not forgotten their L1, and were able to retain the core language competencies to communicate with their family and friends as needed.

Nonetheless, the order of acquisition of the languages does not indicate the survival order. It is often the L1, earliest acquired, that is at risk of loss as the education system takes over. It is also increasingly the case that the L1 is not the third language, so there is no “formal” way to return to it. Sanskrit or a foreign language (French, German, Spanish) or even an official state language occupies this category. The L1, when it is a regional language, is the language that is relegated to limited contexts of use, leading to loss of literacy even if the language is otherwise literary, and eventually becoming a HL. Literacy levels are rarely achieved, and even if acquired, may be lost. It appears that the only way to maintain the L1, then, is via strong ties to the family and the culture. The encouraging fact is that many students denied forgetting a language and, when they did forget one, they were more likely to forget the formal, classroom-learned languages. It was also encouraging that the students valued multilingualism and sought to increase their repertoire. Multilingualism seems to be a desirable and natural state. However, there is a generational gap in how the L1 was used (with older relatives) in comparison to Hindi or English that cannot be denied. English seemed to gain in strength with age and continued education, especially with the unifying experience of entertainment, the internet, and social media use.

We may recall that the participants’ self-assessment of fluency in English tended towards higher levels but with a spread across the scale. The profile questionnaire preceded the test to evaluate English. We note that the English Score Test™ determined that half of the students were at CEFR level C1, which meant they were proficient users of the language, able to perform complex tasks related to work and study. The test also found that about a third were at B2 level, meaning they were independent users of the language with the necessary fluency to communicate without effort. Only about 13% were at levels A1–B1. The self-assessment then did not deviate from the actual assessment. The performance in the test also underscored their self-assessment for reading skills in English over the other languages, the language they used with teachers, and the language in which they studied various subjects in school.

The effect of formal education is visible in the impact it has on the self-assessment of fluency in reading, especially formal reading, in comparison to spoken language. English, again, gains the upper hand for most reading, and students preferred to read any text in English even when it was available in the other languages they knew and were more comfortable with when speaking. While this does not mean that they cannot read if required to do so in Hindi or an L1 (if they have gained literacy in it), the strong preference was for reading in English. In contrast, Hindi showed greater presence in more casual spoken language contexts and within the family and larger society. Here again, it was the L1 that was neglected, showing polarization between those who have forgotten and those who yet remember.

When addressing India’s linguistic diversity and language-centered geography, governance, and education, we need to reconceptualize “heritage” and HL. It can be understood in multiple interconnected ways. First, there is the straightforward historically and culturally important idea of heritage, with the classical languages including Sanskrit, Pali, Prakrit, and the more recently officially recognized classical languages Tamil, Malayalam, Telugu, Kannada, Assamese, Bengali, Odia, and Marathi, which have met the government’s criteria of having (a) high antiquity of their early texts/recorded history over a period of 1,500–2000 years, (b) a body of ancient literature/texts considered valuable heritage by generations of speakers, (c) an original literary tradition not borrowed from another speech community, and (d) a language and literature that are distinct from the modern, with or without discontinuities of form. Second, there is a broader view of heritage in that there are a multitude of (indigenous) languages which, while not “classical”, have long histories of oral traditions and cultures and which play a key role in preserving ethnic, regional, and cultural identities (Devy, 2017). Third, there is the view of heritage having to do with the early acquisition of languages (L1s) which yield to majority languages in the environment and may eventually end up being replaced by them completely in the individual repertoires, but perhaps also within the community, and deals with needs-based bi-/multilingualism.

While the concepts of HL and language attrition are quite widely recognized, how they are manifested and the factors that drive them in India have characteristics that are entirely distinct from the more West-centric discussions. Tied to a few non-official minority languages from *outside* the dominant national sphere, HL is generally discussed in relation to immigrant communities with pressure to assimilate in a nation with a single dominant or societal majority language (e.g., Spanish speakers in the US, Turkish speakers in Germany)^38^ (Guardado, 2002; Fishman, 2001; Polinsky, 2006; Yağmur et al., 1999). Such discussion is focused on 1st, 2nd, and 3rd generation immigrants whose identity is often tied to the ancestral country of origin, where maintenance is directed towards preserving links to that external homeland (Polinsky and Kagan, 2007; Valdés, 2000), and where acquisition is early with declining proficiency with increasing wider societal interactions. Typical causative factors include reduced exposure and the use (Schmid and Köpke, 2017), changing attitudes, and the age of exposure to the dominant language. What is common to both discussions is the L1 as HL, but in the Indian context, HL arises primarily from India’s *internal* and vast linguistic diversity, and the scale is massive (Mohanty, 2006, 2010; Pattanayak, 1990). The L1 could be one of many local languages spoken by communities residing in a region or state where another Indian language is dominant (e.g., Tulu speakers in Kannada-dominant Karnataka); it could be the languages of communities residing in states with another Indian language as the official/dominant one (e.g., Saurashtra speakers in Tamil Nadu); it could be the various tribal and indigenous languages that exist alongside larger regional languages (e.g., Gondi with Marathi in Maharashtra or Irula with Tamil in Tamil Nadu); finally, it could be the (Scheduled) languages of internal migrant communities within India (e.g., Bengali or Odia speakers in Delhi or Mumbai).

Equally, the idea of a dominant language pressuring a heritage language (HL) is multifaceted. It could be the official language of the state, a pan-Indian lingua franca like Hindi or English—especially in formal education and for social mobility—a regional language in certain economic sectors, or a combination of these, creating multiple layers of linguistic pressure. A language can be officially recognized and yet still function as an HL for a particular community in a specific region, where it is not the lingua franca or the language of wider communication. Its official status does not preclude it from being vulnerable to loss. A language’s functionality in different domains heavily influences its vitality and its “heritage” status (Sridhar, 1988). At the individual/family level, too, languages can be HL if their active use is limited to the home in a region dominated by another language. Intergenerational shifts happen extensively within India for many reasons, including internal migration or urbanization without international migration. Linked to all this is linguistic and social identity, which is tied to a specific region, community, or tribe within the nation, and maintenance is about preserving distinct identities within the broader Indian mosaic, often intertwined with regional histories and social structures. The lines between L1, L2, dominant language, and HL are therefore more fluid and contextually determined. A language can be an L1 and an HL simultaneously, facing pressure not from a single dominant “other” language but from a complex hierarchy of languages.

The loss or attrition of L1 (i.e., the HL) in Western contexts follows immersion in and prolonged exposure to a dominant L2 (the national or societal language with social prestige), with a shift from L1 to L2 (Köpke and Schmid, 2004; Schmid, 2011; Schmid and Köpke, 2017). Access to bilingual education and schooling in the dominant language are major contributing factors, and studies focus on specific immigrant languages with attrition in the (often bilingual) individual’s repertoire across immigrant generations (e.g., first-generation fluent, second less so, third may only have receptive knowledge). Such loss of language impacts cultural connections universally.

In the Indian context, attrition can be caused by pressure from multiple dominant languages simultaneously or sequentially—a regional majority language, Hindi, or English—and the specific language exerting pressure varies by region and social context (Annamalai, 2001). As the study shows, one major influence is the presence of and emphasis on Hindi and English in education and for economic opportunities. While individuals may be multilingual, their L1 (HL) erosion follows restrictions on its domains of use. Further, most Indians are often, as we have seen, bi- or multilingual, and attrition indicates the loss or weakening of one language within their multilingual repertoire, rather than a complete shift from L1 monolingualism to L2 monolingualism. This is the common experience of the subjects in the study. Attrition might first manifest in higher formal domains (literacy or complex discourse) while the language is still used for basic interpersonal communication, aligning with research that suggests that internal interfaces are more resilient, while external interfaces where grammar interacts with pragmatics and discourse are more vulnerable to attrition and cross-linguistic influences (Sorace and Serratrice, 2009). Non-reciprocal code-switching (where speakers increasingly rely on the dominant language for lexical items or syntactic structures) can be an indicator of ongoing attrition in the HL (Köpke and Schmid, 2004; Schmid, 2011). We also demonstrated that MoI and policies like the TLF have a profound and complex impact. While aiming for multilingualism, the de facto emphasis in education leads to the attrition of L1s that are not used or supported adequately in the education system, even when they may otherwise have large numbers of speakers. While individual attrition is widespread, as in Western contexts, community-level attrition leading to language endangerment and shift is a critical concern for a vast number of smaller indigenous and tribal languages in India, with a concomitant loss of unique oral traditions and indigenous knowledge systems not documented elsewhere. The pressures are often systemic and affect entire speech communities (Mohanty et al., 2009), but this, as we have pointed out, does not surface directly in this study owing to a lack of representation. Generational shifts are also visible and occur rapidly due to internal migration (rural to urban, state to state), changes in MoI, perceived utility for socio-economic advancement (Vaish, 2008), and urbanization, with a shift from less-valued local dialect/language to a standardized regional language, or from a regional language to English/Hindi.

In the study, many of the L1s are major languages, and the participants can converse and understand their L1 and continue to do so, but they use it in more limited domains with limited vocabulary. The academic or technical domains have been ceded to English/Hindi, and any literacy skills, if gained, are either eroded or lost. Most of the subjects are sequential bilinguals but without any of the socio-cultural pressures to assimilate to the Hindi or English-speaking worlds and without loss of linguistic and/or cultural identities. It has been remarked that HL speakers appear to be different from true “native” speakers in that their pronunciation/phonology or grammar (preferring simpler syntactic structures to complex ones) may differ from that of true monolinguals, and that HL can exert a lasting influence on the languages acquired later (Carreira and Kagan, 2018; Gallo et al., 2021; Gürel, 2008). HL speakers are often referred to as “unbalanced bilinguals” because their competence in the dominant language(s) exceeds their competence in the HL. While this has been contested, it is generally agreed that bilinguals do show domain specialization. The very idea of a fully balanced bilingual may well be an idealization rather than a reality, but we find that a separation of grammatical behaviour and domain-specific lexical specialization needs to be tracked independently. What is noteworthy here is that we find a mixed bag of variable competencies in all the languages known. There can be a marked influence or impact on “accent” (pronunciation and phonology) and the grammar of English or Hindi,^39^ but neither has the L1 been fully replaced, nor have the later languages been fully acquired. We find attrition and loss of skills caused by the direct impact of language policy in education, but we do not find the same kind of forces as in other parts of the world. Of course, there is still a vast array of languages in a very asymmetrical power relationship with the majority languages, even without the impact of the official languages. Some of these languages are already endangered or have fewer than 10,000 speakers and do not even mark a presence in the census, consequently facing socio-cultural marginalization. As we have also seen, those who do not have access to either Hindi or English face socio-economic difficulties and an inability to access HEIs in the technical subjects.

There are, of course, several other constraints at work in India when it comes to languages. Many regional languages are not standardized and do not have a script, which complicates communication as well as development of educational materials, which then prevents their formal use. Urbanization and migration to other states can lead to decline in the use and transmission of indigenous languages or other L1s that are spoken in rural communities. The importance of English both at HEIs and for economic advancement prioritizes learning English, potentially leading to language shifts, attrition, or even complete loss. Educational policies such as the TLF can inadvertently undermine the survival of regional languages, especially when support and exposure are limited. Furthermore, many of India’s endangered languages lack adequate documentation and preservation efforts, increasing the risk of their disappearance as they rely solely on oral transmission (Devy, 2017). Finally, intergenerational language transfer can also be disrupted when parents increasingly use dominant languages like English at home given their own linguistic backgrounds. A decreased presence of L1 in formal education, especially in the later years, is a further impediment to their retention. In essence, while the core linguistic processes of HL maintenance and language attrition are similar, the Indian context amplifies the complexity due to its deep-rooted, widespread multilingualism, its specific socio-political structures concerning language, and the diverse internal pressures shaping language choice and use.

What we learned in this survey is that the primary L1s spoken by the students are predominantly Scheduled languages, recognized by the state and with large speech communities. Yet, we find that the participants report attrition in their language abilities. What the eventual consequences might be for all the L1s remains to be seen. Our main finding in this survey was that the interaction between HL, multilingualism, and formal education policy (TLF) reconfigures the linguistic profiles of individuals and shifts the dominant languages over time, with deleterious consequences for their linguistic ability in L1. The NEP (GOI, 2020) purports to redress the imbalance and restore focus on the L1s, especially in early education, and it remains to be seen whether it will succeed in its aims. In countries like India, where linguistic diversity is a defining characteristic, comprehending the dynamics of HL maintenance and attrition is essential for formulating effective educational programmes and policies that will support linguistic pluralism and cultural heritage. It is debatable whether existing societal multilingualism acts as a buffer against rapid attrition or, conversely, creates more avenues for language shift due to the constant presence and functional necessity of other languages. The scale of internal multilingual contact and the explicit role of language in state and national identity formation create a unique interplay between policy, prestige, and language vitality that shapes both the definition of heritage languages and the processes of attrition.

## Data Availability

Any requests to access the datasets should be directed to the author. Access can only be granted after due institutional approvals and if permissible.

## Footnotes

^1^A new census was delayed by the pandemic and is expected in 2025. Statements 1, 2, 4, and 5 from the census are given.

^2^It is noteworthy that almost 50 MTs are listed under Hindi and contribute to the counts for Hindi. These also include a category ‘Others’ where the populations present less than 10,000 speakers with an unregistered number of varieties.

^3^There is no discussion of multi-dialectal speakers. Many of the variants are grouped under one language and, as such, facts about bi-and multilingualism become hidden by these grouping decisions.

^4^Languages in India belong to four language families, Indo-Aryan (the largest and a branch of the Indo-European family), Dravidian, Austroasiatic, and Tibeto-Burman, with a few isolates.

^5^The highest possible value is 1, which means that no two people have the same mother tongue while the lowest possible value is 0, which says that there is no diversity at all, that everyone has the same mother tongue. The diversity index is computed from the population of each language as a proportion of the total population (UNESCO, 2009).

^6^Interestingly, recent reports seem to show that several states that are part of the Hindi belt (Bihar, Uttar Pradesh, Rajasthan, Himachal) are moving towards monolinguality with 90% of their population saying they speak only one language, Hindi. This needs verification. What has also been noted is that the Human Development Index is higher in states that are multilingual with speakers who are proficient in English. (see https://www.thenewsminute.com/news/90-in-hindi-belt-speak-only-one-language-rest-of-india-more-bilingual-report).

^7^https://www.ef.com/wwen/epi/regions/asia/india/ accessed November 12, 2024.

^8^Part XVII of the Indian Constitution (GOI, 2024), encompassing Articles 343 to 351, addresses official languages. Article 343(1) designates Hindi in Devanagari script as the official language of the Union, while Article 343(2) initially provided for the continued use of English for official purposes for 15 years. The Official Languages Act of 1963 (in Section 3) further ensured the continuation of English alongside Hindi for official purposes and in Parliament. Article 345 allows states to adopt their own official languages. Article 350A mandates instruction in the mother tongue at the primary level, Article 350B provides for a Special Officer for linguistic minorities, and Article 351 focuses on the development of Hindi as a medium of expression for India’s composite culture.

^9^A further complication to this linguistic diversity is the presence of dialectal variation which cuts across socio-economic-geographical and caste differences. Many of the speakers of an L1 are also competent in multiple dialects (see also fn.3) but all L1s are being treated as if they are monolithic. Further, there is also second language learning in the Indian context which has to be nuanced independently. In this study, we are not looking at later learning of languages.

^10^To learn more about the historical context of India’s linguistic policy and the constitutional debates that went into its framing, we refer the reader to Agnihotri (2017). Chandras (2020) offers a general overview of multilingualism in India and Schiffman (1996) examines language policy as a social construct in India and in other nations.

^11^See Koul (2001) for a more generalized survey of L1, Hindi and English across a few states with major and two minority languages. But there are extreme divergences from the data we survey in that Kaul finds a preference for local languages when reading for pleasure and for watching TV/News etc. He also finds that there is no power hierarchy between the L1s and the official languages unlike Mohanty, for example. This may be a function of the passage of time or of the sample he used which may have been inadvertently selected for a positive predisposition to L1.

^12^The complexity of MoI is also mixed in with availability of teachers and their competencies. Rural hiring far exceeds urban. Their impact on language pedagogy is worth studying (see also the AISES 8 report).

^13^Kendriya Vidyalaya, translates directly to ‘Central School’; Jawahar Navodaya (New Beginnings) Vidyalaya are also centrally funded government schools, which cater to socio-economically backward students in rural areas who do not have access to accelerated learning. These schools follow the TLF quite strictly.

^14^National Council of Educational Research and Training (NCERT) sets the curriculum for all school education and creates textbooks which are in wide use, but not obligatory. The state counterpart is the State Council of Educational Research and Training (SCERT) which also creates material and may modify the national curriculum to be more regionally relevant and culturally sensitive.

^15^There are also different ‘boards’ of education. Apart from the boards of each state and the national Central Board of Secondary Education (CBSE) and Council for the Indian School Certificate Examinations (which conducts the ICSE and the ISC exams, what were the earlier Cambridge exams), the IB and IGCSE+A level systems have also made their entry, though primarily in the metros. These are still largely unaffordable schools for most.

^16^The urban–rural is not a uniform divide. Some super big metros will skew the distribution; Delhi for example shows use of English by almost 60%.

^17^Borooah and Sabharwal, (2022) present an analysis of the use of English as MoI.

^18^Pre-school and day care are still reasonably rare phenomena and early child care remains at home. NEP (GOI, 2020) mandates that Grade 1 shall begin only at 6  years, so this will likely change pre-school and Kindergarten experiences.

^19^The gender ratio is typically skewed and no one reported ‘Other’ in the gender category.

^20^English Score Test™ (British Council Division) was administered. This test evaluates vocabulary, grammar, listening and idiomatic usage but is not configured to test free writing or spoken skills. We asked the students to also write a short passage for this reason. Spoken language skills could not be assessed given the large numbers and the time frame within which evaluation needed to be completed.

^21^Over the years, experience has shown that addressing the language needs early aids success in the academic programme (see also Sarma, 2020).

^23^We note the following here: (a) the self-reporting of competencies in English concur with the CEFR scores and written samples though we do not present those correlations here since the main aim of the paper are the changes across the profiles themselves; (b) the self-reporting is quite consistent across cohorts. of students and across various studies, and the aim is to have such comparative analyses available over the next few years.

^24^These are tendencies but do not entail that Hindi is not dominant even when a Dravidian language was listed. Individual school experiences change the trajectory. For example, children in central schools do learn Hindi irrespective of the geographical location.

^25^The languages with the largest number of speakers nationally include Hindi, Bengali, Marathi, Telugu, Tamil, Gujarati, Urdu, Kannada, Odia, Malayalam and map quite straightforwardly to the observed local distribution.

^26^Unlike most other linguistic contexts, Indian institutions are not linguistically uniform – the larger community includes multiple linguistic communities using various languages, both formally and informally, in person and otherwise. It is only within the classroom that there is an MoI.

^27^We must point out that in HEIs in the Indian context the students are dominantly subscribed to an English MoI. As such, it is expected that formal information transactions be in English; poor skill in this context does not mean no skill (unlike in the West)—comprehension is always present, and Romanised Hindi or Hinglish is also commonly used.

^27^We have previous years cohorts who explain their experiences but the data were not collected uniformly and hence, it is not possible to combine them.

^28^See also fn44. We return to this difference in later discussion.

^29^Under the Maharashtra State Board, a second language is compulsory in the last 2 years of school. In these years, students across the country must also elect to pursue one of Science, Humanities or Commerce with associated subject choices. However, for students of science subjects, the language requirement may be replaced by Electronics. This follows from pressures to receive high scores in the final board examination which is a gateway to some HEIs, and a language subject is seen as an impediment to scoring high marks.

^30^Sanskrit does have a large amount of nominal, verbal and other morphology which is quite unlike Hindi, and which in most cases is not successfully learnt at all.

^31^Other inter−/national boards may have only English as the compulsory language. Some states like Tamil Nadu only offer two languages. Under the new National Curriculum Framework (NCERT 2023) students must learn three languages till grade 10 (15 years of age) of which two must be Indian, and two in the last 2 years of school of which one must be Indian. English is not counted among the Indian languages and is seen as a ‘global’ language.

^32^The Expanded Graded Intergenerational Disruption Scale (EGIDS), developed by Lewis and Simons (2010).

^33^Hindi and English are very regularly mixed in what is now called Hinglish. A large amount of vocabulary from English is borrowed into the spoken languages especially with popular words and jargon words. We have noted that children sometimes only know the names of vegetables and fruits, animals etc. in one or the other language and do not have translational equivalents – something we found with true bilinguals (Raghunathan, 2021). A study analysing spoken interactions can throw greater light on this feature.

^34^Montrul (2008) argued that sequential bilinguals developed better linguistic abilities; for example, their lexicon and syntax were better in the HL, especially in spoken language contexts.

^36^It is also worthwhile to note that the preliminary entrance exam is administered in 13 regional languages in addition to Hindi and English, but most choose English. Data reported from 2021 shows us that about 7% of the applicants took the non-English version of the exam and half of those chose Hindi. (https://www.ndtv.com/education/jee-main-2021-over-45000-students-writing-exam-in-regional-languages-2378496) (Accessed March 13, 2025).

^37^This is also of interest. Indian education focuses first on writing. Introduction to the script with spelling and reading take precedence. Unsurprisingly, reading fluency is earlier than the productive skills.

^38^Indian diaspora can be included here.

^39^For example, the use of articles or grammatical gender with agreement: (a) Indian languages do not use definite/indefinite articles and instead use demonstratives or numerals. This feature greatly impacts their use in English in India. (b) Indian languages may mark gender (M-F or M-F-N) or use animacy and gender agreement may be visible on the possessive. This is again a feature that is carried over to English (his_*m_/her_f_ husband_m_) where the possessive shares the gender of the noun (masc) rather than that of the possessor (fem).
