# Supplementary material for: Intersections between heritage, multilingualism, and education: language acquisition in India
Source: Front Hum Neurosci. 2025 Oct 21;19:1538482. doi: 10.3389/fnhum.2025.1538482 (PMC12584054; doi:10.3389/fnhum.2025.1538482)
Supplement: Supplementary file 1 [file Data_Sheet_1.pdf]

Appendix 1: Statement Tables from the 2011 Census (Language) (p6-11)

Images have been reproduced here for the convenience of the reader (p6)

Statement Table 1 from the 2011 Census (Language)

https://language.census.gov.in/eLanguageDivision_VirtualPath/eArchive/pdf/C-16_2011.pdf

STATEMENT-1

ABSTRACT OF SPEAKERS' STRENGTH OF LANGUAGES AND MOTHER TONGUES - 2011

Presented below is an alphabetical abstract of languages and the mother tongues with speakers' strength of 10,000 and above at the all India level, grouped under each language. There are a total of 121 languages and 270 mother tongues. The 22 languages specified in the Eighth Schedule to the Constitution of India are given in Part A and languages other than those specified in the Eighth Schedule (numbering 99) are given in Part B.

PART-A

LANGUAGES SPECIFIED IN THE EIGHTH SCHEDULE (SCHEDULED LANGUAGES)

Name of Language & mother tongue(s) grouped under each language	Number of persons who returned the language (and the mother tongues grouped under each) as their mother tongue)
1	2
**1 ASSAMESE**	1,53,11,351
Assamese	1,48,16,414
Others	4,94,937
**2 BENGALI**	9,72,37,669
Bengali	9,61,77,835
Chakma	2,28,281
Haijong/Hajong	71,792
Rajbangsi	4,75,861
Others	2,83,900
**3 BODO**	14,82,929
Bodo	14,54,547
Kachari	15,984
Mech/Mechhia	11,546
Others	852
**4 DOGRI**	25,96,767
Dogri	25,96,763
Others	4
**5 GUJARATI**	5,54,92,554
Gujarati	5,50,36,204
Gujrao/Gujrau	15,431
Pattani	16,510
Ponchi	13,812
Saurashtra/Saurashtri	2,47,702
Others	1,62,895
**6 HINDI**	52,83,47,193
Awadhi	38,50,906
Baghati/Baghati Pahari	15,835
Bagheli/Baghel Khandi	26,79,129
Bagri Rajasthani	2,34,227
Banjari	15,81,271
Bhadrawahi	98,806
Bhagoria	20,924
Bharmauri/Gaddi	1,81,069
Bhojpuri	5,05,79,447
Bishnoi	12,079
Brajbhasha	15,56,314
Bundeli/Bundel khandi	56,26,356
Chambeali/Chamrali	1,25,746
Chhattisgarhi	1,62,45,190
Churahi	75,552
Dhundhari	14,76,446
Garhwali	24,82,089
Gawari	19,062
Gojri/Gujjari/Gujar	12,27,901
Handuri	47,803
Hara/Harauti	29,44,356
Haryanvi	98,06,519
Hindi	32,22,30,097
Jaunpuri/Jaunsari	1,36,779
Kangri	11,17,342
Khari Boli	50,195
Khortha/Khotta	80,38,735
Kulvi	1,96,295
Kumauni	20,81,057
Kurmali Thar	3,11,175
Lamani/Lambadi/Labani	32,76,548
Laria	89,876
Lodhi	1,39,180
Magadhi/Magahi	1,27,06,825
Malvi	52,12,617
Mandeali	6,22,590
Marwari	78,31,749
Mewari	42,12,262
Mewati	8,56,643
Nagpuria	7,63,014
Nimadi	23,09,265
Padari	17,279
Pahari	32,53,889
Palmuha	23,579
Panch Pargania	2,44,914
Pando/Pandwani	15,595
Pangwali	18,668
Pawari/Powari	3,25,772
Puran/Puran Bhasha	12,375
Rajasthani	2,58,06,344
Sadan/Sadri	43,45,677
Sirmauri	1,07,401
Sondwari	2,29,788
Sugali	1,70,987
Surgujia	17,38,256
Surjapuri	22,56,228
Others	1,67,11,170
**7 KANNADA**	4,37,06,512
Badaga	1,33,550
Kannada	4,35,06,272
Kuruba/Kurumba	24,189
Prakritha/Prakritha Bhasha	12,257
Others	30,244

Statement Table 1 (contd.) from the 2011 Census (Language)

Images have been reproduced here for the convenience of the reader (p7)

https://language.census.gov.in/eLanguageDivision_VirtualPath/eArchive/pdf/C-16_2011.pdf

PART-A *(Contd.)*

LANGUAGES SPECIFIED IN THE EIGHT SCHEDULE (SCHEDULED LANGUAGES)

	Name of Language & mother tongue(s) grouped under each language	Number of persons who returned the language (and the mother tongues grouped under each) as their mother tongue)
	1	2
8	**KASHMIRI**	67,97,587
	Dardi	25,600
	Kashmiri	65,54,369
	Kishtwari	39,748
	Siraji	1,24,896
	Others	52,974
9	**KONKANI**	22,56,502
	Gorboli/Goru/Gorwani	50,259
	Konkani	21,46,906
	Kudubi/Kudumbi	17,209
	Malwani	23,617
	Nawait	13,123
	Others	5,388
10	**MAITHILI**	1,35,83,464
	Maithili	1,33,53,347
	Purbi Maithili	11,116
	Tharu	53,575
	Thati	1,65,420
	Others	6
11	**MALAYALAM**	3,48,38,819
	Malayalam	3,47,76,533
	Pania	22,808
	Yerava	26,563
	Others	12,915
12	**MANIPURI** [1]	17,61,079
	Manipuri	17,60,913
	Others	166
13	**MARATHI**	8,30,26,680
	Are	53,879
	Koli	13,809
	Marathi	8,28,01,140
	Others	1,57,852
14	**NEPALI** [2]	29,26,168
	Nepali	29,25,796
	Others	372
15	**ODIA**	3,75,21,324
	Bhatri	3,34,258
	Bhuiya/Bhuyan[Ori]	32,126
	Bhumijali	34,651
	Desia	2,27,313
	Odia	3,40,59,266
	Proja (Ori)	1,56,354
	Relli	12,969
	Sambalpuri	26,30,381
	Others	34,006
16	**PUNJABI**	3,31,24,726
	Bagri	16,56,588
	Bhateali	23,970
	Bilaspuri Kahluri	2,95,805
	Punjabi	3,11,44,095
	Others	4,268
17	**SANSKRIT**	24,821
	Sanskrit	24,709
	Others	112
18	**SANTALI**	73,68,192
	Karmali	3,58,579
	Mahili	26,399
	Santali	69,73,345
	Others	9,869
19	**SINDHI**	27,72,264
	Bhatia	22,409
	Kachchhi	10,30,602
	Sindhi	16,79,246
	Others	40,007
20	**TAMIL**	6,90,26,881
	Irula/Irular Mozhi	11,870
	Kaikadi	25,870
	Korava	10,421
	Tamil	6,88,88,839
	Yerukala/Yerukula	58,065
	Others	31,816
21	**TELUGU**	8,11,27,740
	Telugu	8,09,12,459
	Vadari	1,98,020
	Others	17,261
22	**URDU**	5,07,72,631
	Bhansari	22,806
	Urdu	5,07,25,762
	Others	24,063

1 Manipuri includes Meithai.
2 Nepali includes Gorkhali.

Statement Table 1 (contd.) from the 2011 Census (Language)

Images have been reproduced here for the convenience of the reader (p8)

https://language.census.gov.in/eLanguageDivision_VirtualPath/eArchive/pdf/C-16_2011.pdf

STATEMENT-1

PART-B

LANGUAGES NOT SPECIFIED IN THE EIGHTH SCHEDULE (NON-SCHEDULED LANGUAGES)

Name of Language & mother tongue(s) grouped under each language	Number of persons who returned the language (and the mother tongues grouped under each) as their mother tongue
1	2
**1 ADI**	2,48,834
Adi	1,10,307
Adi Gallong/Gallong	29,246
Adi Miniyong/Miniyong	13,344
Talgalo	69,256
Others	26,681
**2 AFGHANI/KABULI/PASHTO**	21,677
Afghani/Kabuli/Pashto	21,433
Others	244
**3 ANAL**	27,217
Anal	24,301
Others	2,916
**4 ANGAMI**	1,52,796
Angami	40,721
Others	1,12,075
**5 AO**	2,60,008
Ao	1,19,549
Chungli	70,782
Mongsen	69,094
Others	583
**6 ARABIC/ARBI**	54,947
Arabic/Arbi	54,871
Others	76
**7 BALTI**	13,774
Balti	13,654
Others	120
**8 BHILI/BHILODI**	1,04,13,637
Baori	63,028
Barel	9,91,257
Bhilali	7,53,466
Bhili/Bhilodi	32,06,533
Chodhari	1,10,570
Dhodia	49,097
Gamti/Gavit	1,39,118
Garasia	76,749
Kokna/Kokni/Kukna	4,16,787
Mawchi	98,486
Paradhi	69,085
Pawri	3,11,677
Rathi	47,801
Tadavi	52,347
Varli	3,87,481
Vasava	1,87,036
Wagdi	33,93,991
Others	59,128
**9 BHOTIA**	2,29,954
Bhotia	1,20,583
Bauti	1,00,000
Others	9,371

Name of Language & mother tongue(s) grouped under each language	Number of persons who returned the language (and the mother tongues grouped under each) as their mother tongue
1	2
**10 BHUMIJ**	27,506
Bhumij	10,190
Others	17,316
**11 BISHNUPURIYA**	79,646
Bishnupriya Manipuri/Manipuri Bishnupriya	74,069
Others	5,577
**12 CHAKHESANG**	19,846
Chakhesang	19,846
**13 CHAKRU/CHOKRI**	91,216
Chakru/Chokri	91,216
**14 CHANG**	66,852
Chang	66,852
**15 COORGI/KODAGU**	1,13,857
Kodava	96,918
Coorgi/Kodagu	16,939
**16 DEORI**	32,376
Deori	32,376
**17 DIMASA**	1,37,184
Dimasa	1,33,327
Others	3,857
**18 ENGLISH**	2,59,678
English	2,59,678
**19 GADABA**	40,976
Gadaba	40,965
Others	11
**20 GANGTE**	16,542
Gangte	16,542
**21 GARO**	11,45,323
Garo	11,25,359
Others	19,964
**22 GONDI**	29,84,453
Dorli	47,701
Gondi	28,56,581
Kalari	26,769
Maria/ Muria	15,864
Others	37,538
**23 HALABI**	7,66,297
Halabi	7,65,464
Others	833
**24 HALAM**	38,915
Halam	26,534
Others	12,381

Statement Table 1 (contd.) from the 2011 Census (Language)

Images have been reproduced here for the convenience of the reader (p9)

https://language.census.gov.in/eLanguageDivision_VirtualPath/eArchive/pdf/C-16_2011.pdf

PART-B (*Contd.*)

LANGUAGES NOT SPECIFIED IN THE EIGHTH SCHEDULE (NON-SCHEDULED LANGUAGES)

	Name of Language & mother tongue(s) grouped under each language	Number of persons who returned the language (and the mother tongues grouped under each) as their mother tongue
	1	2
25	**HMAR**	98,988
	Hmar	98,988
26	**HO**	14,21,418
	Ho	14,10,996
	Lohara	10,422
27	**JATAPU**	20,028
	Jatapu	19,990
	Others	38
28	**JUANG**	30,378
	Juang	30,378
29	**KABUI**	1,22,931
	Rongmei	68,706
	Kabui	54,220
	Others	5
30	**KARBI/MIKIR**	5,28,503
	Karbi/Mikir	5,28,503
31	**KHANDESHI**	18,60,236
	Ahirani	16,36,465
	Dangi	1,50,674
	Gujari	57,171
	Khandeshi	10,670
	Others	5,256
32	**KHARIA**	2,97,614
	Kharia	2,93,665
	Others	3,949
33	**KHASI**	14,31,344
	Khasi	10,37,964
	Lyngngam	11,586
	Pnar/Synteng	3,19,324
	War	51,558
	Others	10,912
34	**KHEZHA**	41,625
	Khezha	36,383
	Others	5,242
35	**KHIEMNUNGAN**	61,983
	Khiemnungan	61,968
	Others	15
36	**KHOND/KONDH**	1,55,548
	Khond/Kondh	1,11,693
	Kuvi	43,855
37	**KINNAURI**	83,561
	Kinnauri	83,427
	Others	134
38	**KISAN**	2,06,100
	Kisan	2,06,100
39	**KOCH**	36,434
	Koch	33,962
	Others	2,472
40	**KODA/KORA**	47,268
	Koda/Kora	47,181
	Others	87
41	**KOLAMI**	1,28,451
	Kolami	1,28,451
42	**KOM**	15,108
	Kom	15,108
43	**KONDA** [1]	60,699
	Kodu	32,166
	Konda	24,987
	Others	3,546
44	**KONYAK**	2,44,477
	Konyak	2,44,477
45	**KORKU**	7,27,133
	Korku	6,88,053
	Muwasi	35,827
	Others	3,253
46	**KORWA** [2]	28,453
	Koraku	16,154
	Others	12,299
47	**KOYA**	4,07,423
	Koya	4,07,423
48	**KUI**	9,41,488
	Kui	9,41,377
	Others	111
49	**KUKI**	83,968
	Kuki	82,049
	Others	1,919
50	**KURUKH/ORAON**	19,88,350
	Kurukh/Oraon	19,76,920
	Others	11,430
51	**LADAKHI**	14,952
	Ladakhi	14,952
52	**LAHAULI**	11,574
	Lahauli	11,162
	Others	412
53	**LAHNDA** [3]	1,08,791
	Bahawal Puri	29,253
	Hindi Multani	61,722
	Others	17,816
54	**LAKHER** [4]	42,429
	Mara	38,671
	Others	3,758

Statement Table 1 (contd.) from the 2011 Census (Language)

Images have been reproduced here for the convenience of the reader (p10)

[https://language.census.gov.in/eLanguageDivision_VirtualPath/eArchive/pdf/C-16_2011. pdf](https://language.census.gov.in/eLanguageDivision_VirtualPath/eArchive/pdf/C-16_2011.pdf)

PART-B (*Contd.*)

LANGUAGES NOT SPECIFIED IN THE EIGHTH SCHEDULE (NON-SCHEDULED LANGUAGES)

	Name of Language & mother tongue(s) grouped under each language	Number of persons who returned the language (and the mother tongues grouped under each) as their mother tongue
	1	2
55	**LALUNG**	33,921
	Lalung	33,921
56	**LEPCHA**	47,331
	Lepcha	47,331
57	**LIANGMEI**	49,811
	Liangmei	48,388
	Others	1,423
58	**LIMBU**	40,835
	Limbu	38,067
	Others	2,768
59	**LOTHA**	1,79,467
	Lotha	1,79,467
60	**LUSHAI/MIZO**	8,30,846
	Lushai/Mizo	8,25,900
	Others	4,946
61	**MALTO** [5]	2,34,991
	Kulehiya	75,776
	Pahariya	1,52,814
	Others	6,401
62	**MAO**	2,40,205
	Mao	97,195
	Paola	1,43,001
	Others	9
63	**MARAM**	32,460
	Maram	32,460
64	**MARING**	25,814
	Maring	25,814
65	**MIRI/MISHING**	6,29,954
	Miri/Mishing	6,29,954
66	**MISHMI**	44,100
	Mishmi	15,871
	Others	28,229
67	**MOGH**	36,665
	Mogh	36,652
	Others	13
68	**MONPA**	13,703
	Monpa	13,703
69	**MUNDA**	5,05,922
	Kol	19,868
	Munda	4,64,817
	Others	21,237
70	**MUNDARI**	11,28,228
	Mundari	11,28,050
	Others	178

	Name of Language & mother tongue(s) grouped under each language	Number of persons who returned the language (and the mother tongues grouped under each) as their mother tongue
	1	2
71	**NICOBARESE**	29,099
	Nicobarese	29,099
72	**NISSI/DAFLA**	4,06,532
	Apatani	44,815
	Nissi/Dafla	2,89,166
	Tagin	62,897
	Others	9,654
73	**NOCTE**	30,839
	Nocte	29,810
	Others	1,029
74	**PAITE**	79,507
	Paite	79,443
	Others	64
75	**PARJI** [6]	52,349
	Dhurwa	45,938
	Others	6,411
76	**PAWI**	28,639
	Pawi	28,639
77	**PHOM**	54,416
	Phom	54,416
78	**POCHURY**	21,654
	Pochury	21,568
	Others	86
79	**RABHA**	1,39,986
	Rabha	1,39,985
	Others	1
80	**RAI**	15,644
	Rai	10,427
	Others	5,217
81	**RENGMA**	65,328
	Rengma	65,328
82	**SANGTAM**	76,000
	Sangtam	75,684
	Others	316
83	**SAVARA**	4,09,549
	Savara	4,09,481
	Others	68
84	**SEMA**	10,802
	Sema	10,802
85	**SHERPA**	16,012
	Sherpa	16,012
86	**SHINA**	32,247
	Shina	32,069
	Others	178

Statement Table 1 (contd.) from the 2011 Census (Language)

Images have been reproduced here for the convenience of the reader (p11)

https://language.census.gov.in/eLanguageDivision_VirtualPath/eArchive/pdf/C-16_2011. pdf

PART-B (*Contd.)*

LANGUAGES NOT SPECIFIED IN THE EIGHTH SCHEDULE (NON-SCHEDULED LANGUAGES)

Name of Language & mother tongue(s) grouped under each language	Number of persons who returned the language (and the mother tongues grouped under each) as their mother tongue
1	2
**87 TAMANG**	**20,154**
Tamang	20,154
**88 TANGKHUL**	**1,87,276**
Tangkhul	1,87,263
Others	13
**89 TANGSA** [7]	**38,624**
Tutcha Tangsa	10,234
Others	28,390
**90 THADO**	**2,29,340**
Thado	2,27,114
Others	2,226
**91 TIBETAN**	**1,82,685**
Purkhi	93,500
Tibetan	83,779
Others	5,406
**92 TRIPURI**	**10,11,294**
Kokbarak	9,17,900
Reang	58,539
Tripuri	33,138
Others	1,717
**93 TULU**	**18,46,427**
Tulu	18,41,963
Others	4,464
**94 VAIPHEI**	**42,748**
Vaiphei	42,748
**95 WANCHO**	**59,154**
Wancho	59,154
**96 YIMCHUNGRE**	**83,259**
Chirr	12,300
Tikhir	11,071
Yimchungre	56,538
Others	3,350
**97 ZELIANG**	**63,529**
Zeliang	63,529
**98 ZEMI**	**50,925**
Zemi	50,923
Others	2
**99 ZOU**	**26,545**
Zou	26,545

1.KONDA : A number of Mother tongues including Konda have been grouped together under the language name Konda on the basis of their linguistic affiliation but out of these only Kodu fulfills the criteiron of 10,000 or more speakers at the all-India level and hence only Kodu appears by name as a mother tongue and the rest are included under 'Others' .

2.KORWA: A number of Mother tongues including Korwa have been grouped together under the language name Korwa, on the basis of their linguistic affiliation but out of these only Koraku fulfills the criterion of 10,000 or more speakers at the all-India level and hence only Koraku appears by name as a mother tongue and the rest are included under 'Others'.

3.LAHNDA: A number of Mother tongues including Lahnda have been grouped together under the name Lahnda on the basis of Linguistic Survey of India (LSI) classification of G.A. Grierson, but out of these only Bahwalpuri, Multani and Punchhi fulfils the criterion of 10,000 or more speakers at the all India level and hence Bahawalpuri, Multani and Punchhi appear by name as a Mother tongues and the rest are included under 'Others'. Since the main area of Lahnda speaker is now in Pakistan, India's population of Lahnda speakers is rather small.

4. LAKHER: A number of Mother tongues including Lakher have been grouped together under the language name Lakher, but out of these only Mara fulfills the criterion of 10,000 or more speakers at the all-India level and hence only Mara appears by name as a Mother tongue and the rest are included under 'Others'.

5.MALTO: Malto represents a number of Mother tongues including Malto itself of which only Pahariya fullfills the criterion of 10,000 or more speakers at the all-India level and hence only Pahariya appears by name as a mother tongue and the rest are included under 'Others'. The Malto is used by the people themselves to denote their language and its status as an independent language has been established.

6.PARJI : A number of Mother tongues including Parji have been grouped together under the language name Parji, but out of these only Dhurwa fulfills the criterion of 10,000 or more speakers at the all-India level and hence only Dhurwa appears by name as a Mother tongue and the rest are included under 'Others'.

7. TANGSA : A number of Mother tongues including Tangsa have been grouped together under the language name Tangsa, but out of these only Tutcha Tangsa fulfills the criterion of 10,000 or more speakers at the all-India level and hence only Tutcha Tangsa appears by name as a Mother tongue and the rest are included under 'Others'.

Statement Table 2 from the 2011 Census

Images have been reproduced here for the convenience of the reader (p12)

https://language.census.gov.in/eLanguageDivision_VirtualPath/eArchive/pdf/C-16_2011.pdf

STATEMENT - 2

DISTRIBUTION OF POPULATION BY SCHEDULED AND OTHER LANGUAGES

INDIA, STATES AND UNION TERRITORIES - 2011

India/State/Union Territory#	Scheduled Languages		Other Languages		Total Population 2011	
	Population	Percent	Population	Percent	Population	Percent
1	2	3	4	5	6	7
INDIA	1,17,11,03,853	96.71	3,97,51,124	3.29	1,21,08,54,977	100
1 Jammu & Kashmir	1,21,99,484	97.27	3,41,818	2.73	1,25,41,302	100
2 Himachal Pradesh	67,21,050	97.91	1,43,552	2.09	68,64,602	100
3 Punjab	2,77,03,349	99.86	39,989	0.14	2,77,43,338	100
4 Chandigarh#	10,53,474	99.81	1,976	0.19	10,55,450	100
5 Uttarakhand	1,00,29,461	99.44	56,831	0.56	1,00,86,292	100
6 Haryana	2,53,06,294	99.94	45,168	0.06	2,53,51,462	100
7 NCT of Delhi#	1,67,57,013	99.83	30,928	0.17	1,67,87,941	100
8 Rajasthan	6,48,73,819	94.64	36,74,618	5.36	6,85,48,437	100
9 Uttar Pradesh	19,97,70,172	99.98	42,169	0.02	19,98,12,341	100
10 Bihar	10,38,44,271	99.75	2,55,181	0.25	10,40,99,452	100
11 Sikkim	4,49,632	73.64	1,60,945	26.36	6,10,577	100
12 Arunachal Pradesh	3,85,707	27.87	9,98,020	72.13	13,83,727	100
13 Nagaland	2,34,781	11.87	17,43,721	88.13	19,78,502	100
14 Manipur	16,62,202	58.20	11,93,592	41.80	28,55,794	100
15 Mizoram	1,35,506	12.35	9,61,700	87.65	10,97,206	100
16 Tripura	25,63,639	69.78	11,10,278	30.22	36,73,917	100
17 Meghalaya	4,34,757	14.65	25,32,132	85.35	29,66,889	100
18 Assam	2,89,52,961	92.78	22,52,615	7.22	3,12,05,576	100
19 West Bengal	9,07,93,259	99.47	4,82,856	0.53	9,12,76,115	100
20 Jharkhand	2,97,34,312	90.51	32,53,822	9.49	3,29,88,134	100
21 Odisha	3,87,41,904	91.85	32,32,314	8.15	4,19,74,218	100
22 Chhattisgarh	2,31,62,552	90.68	23,82,646	9.32	2,55,45,198	100
23 Madhya Pradesh	6,72,80,520	92.65	53,46,289	7.35	7,26,26,809	100
24 Gujarat	5,93,80,062	98.25	10,59,630	1.75	6,04,39,692	100
25 Daman & Diu#	2,40,716	98.95	2,531	1.05	2,43,247	100
26 Dadra & Nagar Haveli#	2,14,203	62.32	1,29,506	37.68	3,43,709	100
27 Maharashtra	10,72,93,455	95.43	50,80,878	4.57	11,23,74,333	100
28 Andhra Pradesh	8,38,15,597	99.10	7,65,180	0.90	8,45,80,777	100
29 Karnataka	5,89,40,444	96.47	21,54,853	3.53	6,10,95,297	100
30 Goa	14,41,498	98.83	17,047	1.17	14,58,545	100
31 Lakshadweep#	55,151	85.54	9,322	14.46	64,473	100
32 Kerala	3,32,63,039	99.57	1,43,022	0.43	3,34,06,061	100
33 Tamil Nadu	7,20,98,315	99.93	48,715	0.07	7,21,47,030	100
34 Puducherry#	12,46,854	99.91	1,099	0.09	12,47,953	100
35 Andaman & Nicobar Islands#	3,24,400	85.23	56,181	14.77	3,80,581	100

Statement Table 4 from the 2011 Census

Images have been reproduced here for the convenience of the reader (p15)

https://language.census.gov.in/eLanguageDivision_VirtualPath/eArchive/pdf/C-16_2011.pdf

STATEMENT - 4

SCHEDULED LANGUAGES IN DESCENDING ORDER OF SPEAKERS' STRENGTH - 2011

S. No.	Language	Persons who returned the language as their mother tongue	Percentage to total population
1	2	3	4
1	Hindi	52,83,47,193	43.63
2	Bengali	9,72,37,669	8.03
3	Marathi	8,30,26,680	6.86
4	Telugu	8,11,27,740	6.70
5	Tamil	6,90,26,881	5.70
6	Gujarati	5,54,92,554	4.58
7	Urdu	5,07,72,631	4.19
8	Kannada	4,37,06,512	3.61
9	Odia	3,75,21,324	3.10
10	Malayalam	3,48,38,819	2.88
11	Punjabi	3,31,24,726	2.74
12	Assamese	1,53,11,351	1.26
13	Maithili	1,35,83,464	1.12
14	Santali	73,68,192	0.61
15	Kashmiri	67,97,587	0.56
16	Nepali	29,26,168	0.24
17	Sindhi	27,72,264	0.23
18	Dogri	25,96,767	0.21
19	Konkani	22,56,502	0.19
20	Manipuri	17,61,079	0.15
21	Bodo	14,82,929	0.12
22	Sanskrit	24,821	N

N - Stands for negligible.

Statement Table 5 from the 2011 Census

Images have been reproduced here for the convenience of the reader (p16)

https://language.census.gov.in/eLanguageDivision_VirtualPath/eArchive/pdf/C-16_2011.pdf

STATEMENT - 5

COMPARATIVE SPEAKERS'STRENGTH OF SCHEDULED LANGUAGES - 1971, 1981, 1991,2001 AND 2011

S.No.	Language	Persons who returned the langauge as their mother tongue					Percentage to total population				
		1971	1981	1991	2001	2011	1971	1981	1991	2001	2011
1	2	3	4	5	6	7	8	9	10	11	12
	**India**	**54,81,59,652**	**66,52,87,849**	**83,85,83,988**	**1,02,86,10,328**	**1,21,08,54,977**	**97.14**	**89.23**	**97.05**	**96.56**	**96.71**
1	Hindi*	*20,27,67,971*	*25,77,49,009*	*32,95,18,087*	42,20,48,642	52,83,47,193	36.99	38.74	39.29	41.03	43.63
2	Bengali	4,47,92,312	5,12,98,319	6,95,95,738	8,33,69,769	9,72,37,669	8.17	7.71	8.30	8.11	8.03
3	Marathi	4,17,65,190	4,94,52,922	6,24,81,681	7,19,36,894	8,30,26,680	7.62	7.43	7.45	6.99	6.86
4	Telugu	4,47,56,923	5,06,24,611	6,60,17,615	7,40,02,856	8,11,27,740	8.16	7.61	7.87	7.19	6.70
5	Tamil	3,76,90,106	**	5,30,06,368	6,07,93,814	6,90,26,881	6.88	**	6.32	5.91	5.70
6	Gujarati	2,58,65,012	3,30,63,267	4,06,73,814	4,60,91,617	5,54,92,554	4.72	4.97	4.85	4.48	4.58
7	Urdu	2,86,20,895	3,49,41,435	4,34,06,932	5,15,36,111	5,07,72,631	5.22	5.25	5.18	5.01	4.19
8	Kannada	2,17,10,649	2,56,97,146	3,27,53,676	3,79,24,011	4,37,06,512	3.96	3.86	3.91	3.69	3.61
9	Odia	1,98,63,198	2,30,21,528	2,80,61,313	3,30,17,446	3,75,21,324	3.62	3.46	3.35	3.21	3.10
10	Malayalam	2,19,38,760	2,57,00,705	3,03,77,176	3,30,66,392	3,48,38,819	4.00	3.86	3.62	3.21	2.88
11	Punjabi	1,41,08,443	1,96,11,199	2,33,78,744	2,91,02,477	3,31,24,726	2.57	2.95	2.79	2.83	2.74
12	Assamese	89,59,558	**	1,30,79,696	1,31,68,484	1,53,11,351	1.63	**	1.56	1.28	1.26
13	Maithili	61,30,026	75,22,265	77,66,921	1,21,79,122	1,35,83,464	1.12	1.13	0.93	1.18	1.12
14	Santali	37,86,899	43,32,511	52,16,325	64,69,600	73,68,192	0.69	0.65	0.62	0.63	0.61
15	Kashmiri	24,95,487	31,76,975	#	55,27,698	67,97,587	0.46	0.48	#	0.54	0.56
16	Nepali	14,19,835	13,60,636	20,76,645	28,71,749	29,26,168	0.26	0.20	0.25	0.28	0.24
17	Sindhi	16,76,875	20,44,389	21,22,848	25,35,485	27,72,264	0.31	0.31	0.25	0.25	0.23
18	Dogri	12,99,143	15,30,616	#	22,82,589	25,96,767	0.24	0.23	#	0.22	0.21
19	Konkani	15,08,432	15,70,108	17,60,607	24,89,015	22,56,502	0.28	0.24	0.21	0.24	0.19
20	Manipuri $	7,91,714	9,01,407	12,70,216	14,66,705	17,61,079	0.14	0.14	0.15	0.14	0.15
21	Bodo	5,56,576	**	12,21,881	13,50,478	14,82,929	0.10	**	0.15	0.13	0.12
22	Sanskrit	2,212	6,106	49,736	14,135	24,821	N	N	0.01	N	N

Note:

1. The percentage of speakers of each language for 1981 has been worked out on the total population of India excluding the population of Assam where the 1981 Census was not conducted due to disturbed conditions
2. Full figures for Tamil, Assam and Bodo for 1981 are not available as the census records for Tamil Nadu were lost due to floods and the 1981 Census could not be conducted in Assam due to the disturbed conditions then prevailing there. Therefore, percentage to total population of Tamil and Assamese are not given.
3. The percentage of speakers of each language for 1991 has been worked out on the total population of India excluding the population of Jammu & Kashmir where the 1991 Census was not conducted due to disturbed conditions.
4. The percentage of speakers of each language for 2001 has been worked out on the population of India excluding the population of Mao-Maram, Paomata and Purul subdivisions of Senapati district of Manipur due to cancellation of census results over there.

Full figures for Kashmiri & Dogri language for 1991 are not available as the 1991 Census was not conducted in Jammu & Kashmir due to disturbed conditions.

$ Excludes figures of Paomata, Mao-Maram and Purul sub-divisions of Senapati district of Manipur for 2001.

N' - Stands for Negligible.

@ Maithili figure has been extracted from Hindi language from 1971 census to 1991 census since it was one of the mother tongues grouped under Hindi during the period.
