# Supplementary material for: Intersections between heritage, multilingualism, and education: language acquisition in India
Source: Front Hum Neurosci. 2025 Oct 21;19:1538482. doi: 10.3389/fnhum.2025.1538482 (PMC12584054; doi:10.3389/fnhum.2025.1538482)
Supplement: Supplementary file 2 [file Data_Sheet_2.pdf]

Appendix 2

Adapted LEAP Questionnaire (used in the study)
Instructions: *This questionnaire allows us to learn about your language experiences. We look at exposure to different languages, how they are used, your fluency, and medium of instruction to plan courses and to develop resources at the Language Centre.*
Disclosure: *Informed Consent*
Q1-3. Personal details collected; e-Pmail, gender, roll number.
Q4. Please list all the languages you know — **in order of dominance** (meaning, the language you use the most to the least; it may be 2, 3 or 4 — doesn't matter).
Q 5-7: *The percentages should add to 100!*
Q5. What **percentage of the time** (on average) are you currently exposed to and use these languages? Language 1 is the one that is dominant, Language 2 next and so on.
Q6. If a text (article, story) is available in all the languages you know, in what percentage of the cases would you choose to **read** it in **each of the languages** you know?
Q7. If you are speaking to someone who knows **all** the languages you know, what percentage of the time would you choose to speak **each of the languages** you know?
Q8. For all the languages you listed above, please place them **in order of acquisition or learning** (start with your native or mother tongue first). If you acquired more than one language equally, you may say so.
Q9. What was the language in which subjects (like History, Geography and Science) were taught in your school (till Std. 10)?
Q10. What was the language you used most frequently with teachers?
Q11. What was your third language in school?
Q12. Have you forgotten any language that you used to speak? Say which.
Q13. If we wish to find out more about your language experience, can we contact you? Y/N

Individual Language Use: The same questions were asked in each of the three subsections. *In each subsection below, we will ask you about English, Hindi, and Regional Language 1 (your mother or other tongue) which you use/have used. Please answer carefully after thinking about your language use.*
Q14. Age you began acquiring/learning it
Q15. Age you became fluent speaker
Q16. Age you could start reading
Q17. Age you became a fluent reader
Q18. On a scale of 1-10, how would you rate your fluency in speaking and holding a conversation in all situations? (Scale: Low Fluency – Native Speaker)
Q19. On a scale of 1-10, how would you rate your fluency in reading a text, signs, instructions, filling out forms etc.? (Scale: Very slow reader – Can read rapidly)
Q20. Choose the contexts in which you use the language, check as many as apply.
Interacting with family - especially older members, grandparents

a) Interacting with family - brothers/sisters, parents, younger cousins etc.
b) Interacting with friends
c) Reading - casual
d) Reading - formal
e) Watching TV programmes, movies, listening to the news etc.
f) Social media - Instagram, WhatsApp, etc.
g) Music
h) Shopping, vendors, larger society - neighbours and others
i) Other
